# Vitamin D Modulation of Mitochondrial Oxidative Metabolism and mTOR Enforces Stress Adaptations and Anticancer Responses

**DOI:** 10.1002/jbm4.10572

**Published:** 2021-12-01

**Authors:** Mikayla Quigley, Sandra Rieger, Enrico Capobianco, Zheng Wang, Hengguang Zhao, Martin Hewison, Thomas S Lisse

**Affiliations:** ^1^ Biology Department University of Miami Coral Gables FL USA; ^2^ Dana Farber Cancer Institute Boston MA USA; ^3^ Sylvester Comprehensive Cancer Center, Miller School of Medicine University of Miami Miami FL USA; ^4^ Institute for Data Science and Computing University of Miami Coral Gables FL USA; ^5^ Department of Computer Science University of Miami Coral Gables FL USA; ^6^ Department of Dermatology The First Affiliated Hospital of Chongqing Medical University Chongqing China; ^7^ Institute of Metabolism and Systems Research University of Birmingham Birmingham UK

**Keywords:** BONE, CANCER, CYP24A1, DDIT4, METABOLISM, MG‐63, MITOCHONDRIA, OSTEOBLAST, OSTEOSARCOMA, REDD1, ROS, SOD, SOD1, SOD2, STRESS, TUMOR, UNFOLDED PROTEIN RESPONSE, VDR, VITAMIN D, VITAMIN D DEFICIENCY, VITAMIN D RECEPTOR

## Abstract

The relationship between the active form of vitamin D_3_ (1,25‐dihydroxyvitamin D, 1,25(OH)_2_D) and reactive oxygen species (ROS), two integral signaling molecules of the cell, is poorly understood. This is striking, given that both factors are involved in cancer cell regulation and metabolism. Mitochondria (mt) dysfunction is one of the main drivers of cancer, producing more mitochondria, higher cellular energy, and ROS that can enhance oxidative stress and stress tolerance responses. To study the effects of 1,25(OH)_2_D on metabolic and mt dysfunction, we used the vitamin D receptor (VDR)‐sensitive MG‐63 osteosarcoma cell model. Using biochemical approaches, 1,25(OH)_2_D decreased mt ROS levels, membrane potential (ΔΨ_mt_), biogenesis, and translation, while enforcing endoplasmic reticulum/mitohormetic stress adaptive responses. Using a mitochondria‐focused transcriptomic approach, gene set enrichment and pathway analyses show that 1,25(OH)_2_D lowered mt fusion/fission and oxidative phosphorylation (OXPHOS). By contrast, mitophagy, ROS defense, and epigenetic gene regulation were enhanced after 1,25(OH)_2_D treatment, as well as key metabolic enzymes that regulate fluxes of substrates for cellular architecture and a shift toward non‐oxidative energy metabolism. ATACseq revealed putative oxi‐sensitive and tumor‐suppressing transcription factors that may regulate important mt functional genes such as the mTORC1 inhibitor, *DDIT4/REDD1*. DDIT4/REDD1 was predominantly localized to the outer mt membrane in untreated MG‐63 cells yet sequestered in the cytoplasm after 1,25(OH)_2_D and rotenone treatments, suggesting a level of control by membrane depolarization to facilitate its cytoplasmic mTORC1 inhibitory function. The results show that 1,25(OH)_2_D activates distinct adaptive metabolic responses involving mitochondria to regain redox balance and control the growth of osteosarcoma cells. © 2021 The Authors. *JBMR Plus* published by Wiley Periodicals LLC on behalf of American Society for Bone and Mineral Research.

## 1 Introduction

The altered metabolism of cancer cells imparts a large impact on redox (reduction–oxidation) homeostasis, resulting in enhanced production of reactive oxygen species (ROS) that drives and sustains cancer cell proliferation and evasion.^(^ ^1^ ^)^ One counter measurement to elevated ROS is the increase of antioxidants, which allows cancer cells to persist in a damaging environment. However, as cancer cells persist in the continuing pro‐oxidant environment, DNA is further damaged and the genome becomes more unstable with concomitant metabolic reprogramming.^(^ ^1^ ^)^ Over time, the metabolic reprogramming further distances cancer cells from redox homeostasis, leading to a vicious cycle of accumulating genetic lesions toward cancer progression. Understanding the mechanisms and how to control the dysfunctional and dysregulated metabolism of cancer cells is a major challenge in cancer biology.

Vitamin D consists of two major forms known as vitamin D_2_ (ergocalciferol) and vitamin D_3_ (cholecalciferol) that are most known for their roles in the maintenance of mineral homeostasis and growth of the body.^(^ ^2^ ^)^ In humans, vitamin D_3_ is synthesized in the skin upon UVB exposure, whereby vitamin D_2_ is obtained from plant sources in our diets. Both forms of vitamin D are biologically inert and require conversion and hydroxylation to 25(OH)D by vitamin D‐25‐hydroxylase in the liver, the major measurement of one's vitamin D status.^(^ ^3^ ^)^

25(OH)D is further hydroxylated in the kidneys or within specialized cell types by the 25(OH)D‐1‐OHase (CYP27B1) to yield the biologically active metabolite of vitamin D, 1‐alpha, 25‐dihydroxyvitamin D (also called 1,25(OH)_2_D).^(^ ^2^, ^4^, ^5^, ^6^, ^7^, ^8^ ^)^ Circulating 1,25(OH)_2_D effects are mediated by the vitamin D receptor (VDR), a member of the intracellular nuclear receptor superfamily.^(^ ^9^ ^)^ Perturbation of the vitamin D metabolic and signaling systems in humans and animals are associated with several diseases and disorders, including alopecia,^(^ ^10^, ^11^ ^)^ osteosarcopenia,^(^ ^12^ ^)^ diabetes,^(^ ^13^ ^)^ and cancer.^(^ ^2^ ^)^ A large body of studies have all implicated a suppressive role of vitamin D in cancer development and improved cancer patient and animal survival.^(^ ^2^, ^14^, ^15^, ^16^, ^17^ ^)^ For example, elevated serum 25(OH)D levels at diagnosis have been linked to extended survival rates in cancer patients.^(^ ^18^ ^)^ And most recently, a large clinical trial involving vitamin D_3_ supplementation suggests that cholecalciferol can benefit patients with advanced or lethal cancers by decreasing mortality and prolonging survival in the study population.^(^ ^15^, ^19^ ^)^ Despite these encouraging results from the clinical trial, the precise mechanism for anticancer effects of vitamin D and its metabolites remains elusive.

Understanding how what we eat (e.g., fruits and vegetables, which are rich in antioxidants like vitamin C^(^ ^20^ ^)^), what we expose ourselves to (e.g., sunlight, pollution), and the metabolism that can lead to energy‐reducing equivalents that drive cellular replication and differentiation at the genetic level provides insight toward the circle of life. In contrast, understanding how molecular factors such as ROS and their antioxidants can regulate the cell cycle, differentiation, and adaptive responses (e.g., DNA damage, mutagenesis) at the genetic level provides insight toward the circle of death. Numerous mechanisms exist to help dictate the consequences of cellular oxidative stress. For example, the cytoprotective upregulation of DNA repair transcripts allow for DNA mismatch repair, non‐homologous end‐joining, and base excision repair in cells to handle the high levels of oxidative damage. Other avenues may consist of oxidation of membrane receptors, signaling molecules, redox‐sensitive transcription factors, and epigenetic transcriptional regulators, including histone deacetylase family members to regulate the cell's response to stress and cancer development.^(^ ^21^ ^)^ Understanding the metabolic oxidation/reduction reactions and cellular responses to various biological and environmental factors remains a major milestone in cancer biology and therapy.

Thus, in the present study, we postulated that effects on the metabolic oxidation/reduction reactions that are characteristic of cancer cells may be crucial to the anticancer effects of the environmental/nutritional factor 1,25(OH)_2_D (also called calcitriol). We applied genomewide transcriptomic and epigenomic approaches to provide a comprehensive understanding of how 1,25(OH)_2_D modulates mitochondrial functions and counteracts tumorigenicity within the MG‐63 osteosarcoma cell model, followed by mitochondrial biochemical and ultrastructural investigations. Based on these approaches, we provide evidence of novel regulators of mitochondrial stress, biogenesis, translation, organellar hormesis, and cancer metabolic fates mediated by 1,25(OH)_2_D, and cooperating factors leading to an overall reduction in oxidative stress levels. In particular, although the tumor suppressor DNA damage–inducible transcript 4 (DDIT4) is induced under stress conditions in normal bone cells to inhibit metabolism activated by the mammalian target of rapamycin (mTOR) in the cytoplasm,^(^ ^22^ ^)^ MG‐63 osteosarcoma cells exhibit high levels of DDIT4 sequestered to the mitochondria as a potential mechanism to regulate mTOR activation and cancer progression. In contrast, 1,25(OH)_2_D treatment of MG‐63 cells increased the expression and cytoplasmic localization of DDIT4 through separation from the outer mitochondrial membrane. Ironically, a meta‐analysis of numerous cancer cell types identified DDIT4 as being overexpressed compared with non‐cancerous cells and associated with poor survival outcomes despite being a potent mTOR inhibitor.^(^ ^23^ ^)^ Based on our findings from MG‐63 cells, we propose that 1,25(OH)_2_D may suppress tumor progression of other cancer types that involves mitochondrial‐to‐cytoplasmic DDIT4/REDD1 exchange. Overall, the results herein establish that 1,25(OH)_2_D can target the deregulation of specific metabolic hubs within osteosarcoma cells to suppress tumorigenicity, thus attempting to maintain the delicate balance between the cycle of life and death.

## 2 Materials and Methods

### 2.1 Reagents and cell culture

Crystalline 1,25(OH)_2_D (679101, MilliporeSigma, Burlington, MA, USA) and the vitamin D receptor antagonist ZK159222 (VAZ, Toronto Research Chemicals, Toronto, Canada) were reconstituted in ethanol and kept at −80°C. Human MG‐63 osteosarcoma cells (CRL‐1427; American Type Culture Collection, Manassas, VA, USA) were cultured in complete media containing Eagle's minimum essential medium (ATCC, 30–2003), 10% heat‐inactivated fetal bovine serum (Gibco, Thermo Fisher Scientific, Waltham, MA, USA), and 100 U/mL penicillin, 100 mg/mL streptomycin (Life Technologies, Carlsbad, CA, USA). For assays, cells were treated with 0 (vehicle; equal‐volume ethanol; 0.0001%), 10 nM, and 100 nM 1,25(OH)_2_D incubated in tissue culture plates (CytoOne, USA Scientific, Ocala, FL, USA) at 37°C in a humidified atmosphere of 5% CO_2_, 95% air.

### 2.2 Soft agar colony formation assay

MG‐63 cells (1000 per well, 24‐well plate) were seeded into 0.4% low‐melting‐point agarose (Lonza, Basel Switzerland; 50101) on top of a 1% agarose layer. Cells were maintained in a 5% CO_2_ incubator at 37°C for approximately 14 days with vehicle or vitamin D. Colonies were fixed in methanol and stained with crystal violet. For quantification, crystal violet‐positive colonies were counted using a dissecting scope (Zeiss Stereo 305, Carl Zeiss, Jena, Germany) with the ImageJ software. All assays were set up in five to six replicates per condition. A one‐way ANOVA test was performed with Tukey's multiple comparisons test.

### 2.3 RNA sequencing and functional/pathway/gene set enrichment analyses

Cell preparations (*n* = 2 per treatment condition) were collected and total RNA was purified using the PureLink RNA Mini kit (Thermo Fisher Scientific, 12183018A) with DNase set (Thermo Fisher Scientific, 12185010). RNA quality was tested by Agilent (Santa Clara, CA, USA) Bioanalyzer and confirmed to have RIN numbers >8.5. Library preparation and RNA‐sequencing were performed at the Oncogenomics Core Facility at the Sylvester Comprehensive Cancer Center (University of Miami). Samples were sequenced using 75 bp paired ends with an Illumina (San Diego, CA, USA) NextSeq 500 generating reads of ∼30 million per sample that were trimmed and filtered using Cutadapt. Compressed Fastq.gz files were uploaded to a Galaxy account (https://usegalaxy.org/),^(^ ^24^ ^)^ and data sets were concatenated tail‐to‐head (Galaxy Version 0.1.0). A MultiQC analysis was performed on all FastQC raw data files to generate a summarized QC report. HISAT2 was performed for sample alignment to the human genome (Ensembl 87: GRCh38.p7 human transcriptome) to generate BAM files with the most reads (80% to 90%) aligning successfully. Samstools stat was performed for further quality control of BAM files, and HTseq‐count was performed to generate non‐normalized gene counts for each sample. Normalization of RNAseq gene counts (counts per million [CPM]), and differential gene expression and visualization analyses were performed with iDEP (http://bioinformatics.sdstate.edu/idep93). Differentially expressed genes were determined using DESeq2 with the false discovery rate (FDR) set to 0.05 and logFC (1) compared with controls. For individual gene expression plots derived from RNAseq, a two‐way ANOVA was performed with Bonferroni's multiple comparisons test using the CPM values, where the *p* value summaries were depicted as ^****^ *p* ≤ 0.0001, ^***^ *p* ≤ 0.001, ^**^ *p* ≤ 0.01, and ^*^ *p* ≤ 0.05. Gprofiler was utilized to perform gene name conversion (ENTREX TO ENTRZ) and basic functional annotation analyses (http://biit.cs.ut.ee/gprofiler/gost). To adjust for the FDR, we only considered terms with a Benjamini–Hochberg adjusted *p* value of 0.05. iDEP was utilized for the distribution of transformed data and the generation of scatter plots of sample correlations, hierarchical and k‐means heatmap generation, and pathway analyses. GSEA and GAGE were performed using statistically significant differentially expressed genes to determine whether a priori defined set of genes were different between the two biological states. For GSEA and GAGE, the molecular Signatures Database v7.3 with the hallmark and canonical (KEGG) gene sets were applied.

### 2.4 ATAC‐Seq and data analysis

Cells from two independent experiments were collected and open chromatin was assessed using an ATACseq kit (Active Motif, Carlsbad, CA, USA; 53150). DNAseq library preparation was completed at the Oncogenomics Core Facility at the Sylvester Comprehensive Cancer Center. Samples were sequenced using 100‐bp paired ends with an Illumina NovaSeq 6000. Compressed Fastq.gz files were uploaded to a Galaxy account (https://usegalaxy.org/), concatenated, and subsequent sequencing reads (∼40 million per sample) were trimmed off the Nextera adapter sequences and filtered using Cutadapt. Reads were mapped to the reference genome (hg38 Canonical) using Bowtie2 with presets “very sensitive end‐to‐end (–very‐sensitive),” “set the maximum fragment length for valid paired‐end alignments: 1000,” and allowing mate dovetailing to generate BAM files. We filtered uninformative reads with low mapping quality and were not properly paired using Filter BAM data sets on a variety of attributes (Galaxy Version 2.4.1). Filter on read mapping quality (phred scale) was set to ≥30. ATACseq motif discovery was conducted using HOMER (http://www.homer.ucsd.edu).

### 2.5 Multi‐omics analysis of genes that encode mitochondrial proteins

We examined the differential expression of genes that encode mitochondria‐related proteins based on a compendium from MitoCarta (https://www.broadinstitute.org/mitocarta) and mitoXplorer (http://mitoxplorer.ibdm.univ-mrs.fr). We appraised mitochondrial protein‐encoding genes using Venn analysis (http://www.interactivenn.net) between the mitochondrial compendium and the differentially regulated genes derived from our RNAseq studies.

### 2.6 Quantitative real‐time RT‐PCR (qPCR) and analysis

RNA was prepared like the RNAseq studies. cDNA was synthesized using 200 ng total RNA with the ProtoScript First Strand cDNA Synthesis kit (New England Biolabs, Ipswich, MA, USA) utilizing random hexamers. All cDNAs were amplified under the following conditions: 95°C for 10 minutes to activate AmpliTaq Gold Polymerase, followed by 40 cycles of 95°C for 15 seconds and 60°C for 1 minute with an internal ROX reference dye. qPCR analysis was performed on a QuantStudio 3 Real‐Time instrument (Thermo Fisher Scientific) utilizing the Power SYBR Green PCR Master mix (Thermo Fisher Scientific; Supplemental Table S1). Target genes were normalized to beta actin mRNA expression. For the primer design, the human genome sequence coverage assembly GRCh38.p13 was utilized from the Genome Reference Consortium. Data were presented as fold induction of treatments compared with 0 nM (vehicle) normalized to beta actin mRNA levels (i.e., the comparative CT Livak method). Melting curve analysis was performed for all primer sets to eliminate those that yielded primer‐dimers. The *p* values reflect the log fold‐change compared with the vehicle (0 nM) condition (*n* = 3 experimental samples ± SD). A two‐way ANOVA test with Sidak's multiple comparisons test was performed between vehicle and treatment data sets using Prism (GraphPad, La Jolla, CA, USA) where the *p* value summaries were depicted as ^****^ *p* ≤ 0.0001, ^***^ *p* ≤ 0.001, ^**^ *p* ≤ 0.01, and ^*^ *p* ≤ 0.05.

### 2.7 MitoSOX red mitochondrial superoxide indicator and live‐cell Apotome imaging

MitoSOX Red reagent (Thermo Fisher Scientific, M36008) was used to detect mitochondrial superoxide levels in live cells. MG‐63 cells were cultured in Millicell EZ chamber slides (EMD Millipore). A 5 mM MitoSOX Red reagent stock solution was made by dilution into dimethyl sulfoxide (DMSO). A 5 μM MitoSOX Red reagent working solution was made by diluting the stock into a culture medium. Cells were loaded with MitoSOX Red reagent by incubating for 10 minutes at 37°C protected from light. Hoechst 33342 (1:2000) live‐cell dye was used as a counterstain to detect the nuclei of live cells (Thermo Fisher Scientific, H1399). Cells were washed three times with a warm medium. Intensity measurements were obtained using the Zen Blue software (Zeiss) analyzed using Prism 8 (GraphPad). Rotenone (Sigma, St. Louis, MO, USA; R8875) was applied as a positive control. For each replicate (*n* = 6 replicates/condition), average ratios were derived from four different fields of views of 5 to 10 individual cells. Data are presented as mean ± SEM error bars; ^****^ *p* ≤ 0.0001, ^***^ *p* ≤ 0.001, ^**^ *p* ≤ 0.01, and ^*^ *p* ≤ 0.05 (one‐way ANOVA with Tukey's multiple comparisons test compared with vehicle).

### 2.8 Mitochondrial membrane potential (ΔΨ_M_ ) measurements and live‐cell Apotome imaging

A JC‐1 (5,5,6,6′‐tetrachloro‐1,1′,3,3′ tetraethylbenzimi‐dazoylcarbocyanine iodide) mitochondrial membrane potential detection kit (Biotium, Fremont, CA, USA; 30001) was used to measure mitochondrial membrane potential changes in live cells. MG‐63 cells were cultured in Millicell EZ chamber slides (EMD Millipore). All experiments were performed in a low‐light setting. A 1× working solution of JC‐1 dye was prepared in a cell culture medium, and cells were incubated in a 37°C cell culture incubator for 15 minutes. Cells were washed once with PBS and replenished with a fresh culture medium. Hoechst 33342 live‐cell dye was used to detect the nuclei of live cells (Thermo Fisher Scientific, H1399). Cells were observed immediately using a Zeiss Observer 7 ApoTome2 microscope using a dual band‐pass filter designed to simultaneously detect fluorescein and rhodamine, or fluorescein and Texas Red. The spectral properties of the JC‐1 dye consist of Excitation/Emission (cytoplasm): 510/527 nm (green) and Excitation/Emission (polarized mitochondria): 585/590 nm (red). The ellipsoid spot measurement tool in Imaris was used to select perinuclear JC‐1‐labeled mitochondria to determine JC‐1 aggregate:monomer intensity ratios. The spot intensity tool (Imaris) was used to determine the intensity (*y* axis) and position (*x* axis) between spot‐to‐spot (i.e., a series of mitochondria‐to‐mitochondria) in treated cells. For each replicate (*n* = 6–8 replicates/condition), average ratios were derived from four different fields of views of 6 to 8 individual cells. Data are presented as mean ± SEM error bars; ^****^ *p* ≤ 0.0001, ^***^ *p* ≤ 0.001, ^**^ *p* ≤ 0.01, and ^*^ *p* ≤ 0.05 (two‐way ANOVA with Sidak's multiple comparisons test compared to vehicle).

### 2.9 Mitochondrial biogenesis in‐cell ELISA assay

Mitochondrial biogenesis and translation were monitored in MG‐63 cells using an in‐cell ELISA kit (Abcam, Cambridge, MA, USA; Ab110217). In brief, MG‐63 cells were cultured in collagen‐coated 96‐well plates and treated with 1,25(OH)_2_D at various concentrations and durations in five replications. After the treatment series, cells were fixed with 4% PFA and then quenched for endogenous alkaline phosphatase activity using acetic acid. Primary antibody cocktails containing COX‐1 and SDHA recognizing antibodies were added to the wells. Afterward, secondary HRP and AP‐conjugated antibodies were applied and detected using a microplate reader at OD 405 nm (for AP detection of SDHA) and 600 nm (for HRP detection of COX‐1). Measurements were normalized to the Janus Green staining intensity at OD 595 nm to account for differences in cell seeding.

### 2.10 Immunofluorescence labeling and analysis of MG‐63 cells

MG‐63 cells were cultured in Millicell EZ chamber slides (EMD Millipore) and fixed in either 80% methanol or 4% paraformaldehyde (PFA) in 0.1 M phosphate buffer (PBS, pH 7.4) for 10 minutes. PFA fixed cells were permeabilized with 0.2% Triton X‐100 in PBS for 5 to 15 minutes at room temperature, followed by washes with PBS. Cells were blocked with normal horse/goat serum for non‐specific background and then incubated with primary antibodies at a 1:200 dilution for 1 hour at room temperature. Primary antibodies used in this study included rabbit monoclonal to VDAC1 (Abcam, ab154856), and rabbit monoclonal to REDD1/DDIT4 (Abcam, ab191871). After washing steps in phosphate‐buffered saline‐Tween‐20, the cells were incubated at room temperature for 20 minutes with corresponding species‐specific secondary antibodies (Alexa series at 1:2000, Life Technologies). The slides were covered with Vectashield medium containing 4′,6‐diamidino‐2‐phenylindole (DAPI; Vector Laboratories, Burlingame, CA, USA; H‐1200‐10) for nuclei staining and then mounted with a glass coverslip. Negative controls were included that had either no primary or secondary antibodies in the blocking buffer. Immunofluorescence confocal‐like microscopy was performed using a Zeiss Observer 7 ApoTome2 system. We carefully selected the wavelength ranges, emission filters, and dichroic mirrors to avoid signal bleed‐through. Deconvolution and Apotome processing (i.e., extended depth of view) of stacked images was performed using the ZenBlue software (Zeiss Microscopy). Image stacks were reconstructed and visualized as three‐dimensional (3D) volumes with Imaris software (Bitplane, Zurich, Switzerland). The Imaris Spot detection algorithm was used as described by the manufacturer for semiautomatic identification and counting of fluorescently labeled mitochondria and cytoplasmic components. Means of expression intensity of mitochondrial and cytoplasmic regions within individual cells were compared between vehicle and 1,25(OH)_2_D‐treated samples. For analysis, individual experiments (*n* = 4) were performed whereby each experiment entailed an assessment of 4 to 6 individual sets of cells for technical replication. For some experiments, Imaris (Bitplane) and MATLAB were used to generate 3D rendered models of protein expression and colocalization. Spots are located at the local maxima of the filtered image with background subtraction. Imaris calculated a “spot quality” (minimum of 100) based on intensity differences and shapes for spot rendering and was adjusted to include the signal of interest. Colocalization analysis was performed using the “spot” tool to designate the distance threshold and the mean distance between the “VDAC” and “DDIT4” colocalized spots. A two‐way ANOVA test with Sidak's multiple comparisons test was performed between vehicle and treatment data sets using Prism (GraphPad) where the *p* value summaries were depicted as ^****^ *p* ≤ 0.0001, ^***^ *p* ≤ 0.001, ^**^ *p* ≤ 0.01, and ^*^ *p* ≤ 0.05. Statistical significance was accepted at ^*^ *p* ≤ 0.05.

### 2.11 Transmission electron microscopy (TEM)

TEM was performed at the Transmission Electron Microscopy Core Facility at the Miller School of Medicine, University of Miami. The TEM Core prepared the cells for electron microscopy and performed embedding and semi‐thin (1 μm) and thin (100 nm) sectioning of the samples and final imaging with a JEOL JEM‐1400 electron microscope. For analysis, 7 to 10 cells were investigated per condition, in which we averaged parameters between 20 to 40 mitochondria per cell.

### 2.12 Stimulation and measurement of ER stress

Known ER stress inducers tunicamycin (Sigma‐Aldrich, T7765) and thapsigargin (Sigma‐Aldrich, T9033) were diluted in ethanol and exposed to cells for 6 hours with appropriate vehicle controls. Both endpoint semiquantitative and quantitative real‐time PCR methods were used to assess ER stress based on Yoon Seung‐Bin and colleagues^25^ with adjustments (e.g., the annealing temperature of 62°C was used instead). For the endpoint PCR reaction, the Phusion DNA polymerase (Thermo Fisher Scientfic) was used, and a 2.5% agarose gel was utilized to assess ER stress PCR products. u/s/tXBP1 primers relative to the 26 bp of XBP1 removed by IRE1 were used for real‐time PCR reactions (Supplemental Table S1). Housekeeping genes (Gapdh, 18sRNA) and the total amount of XBP1 (Supplemental Table S1) were used to normalize gene expression.

## 3 Results

### 3.1 Genomewide assessment of 1,25(OH) _2_D‐mediated transcription using RNAseq

**Fig 1:**
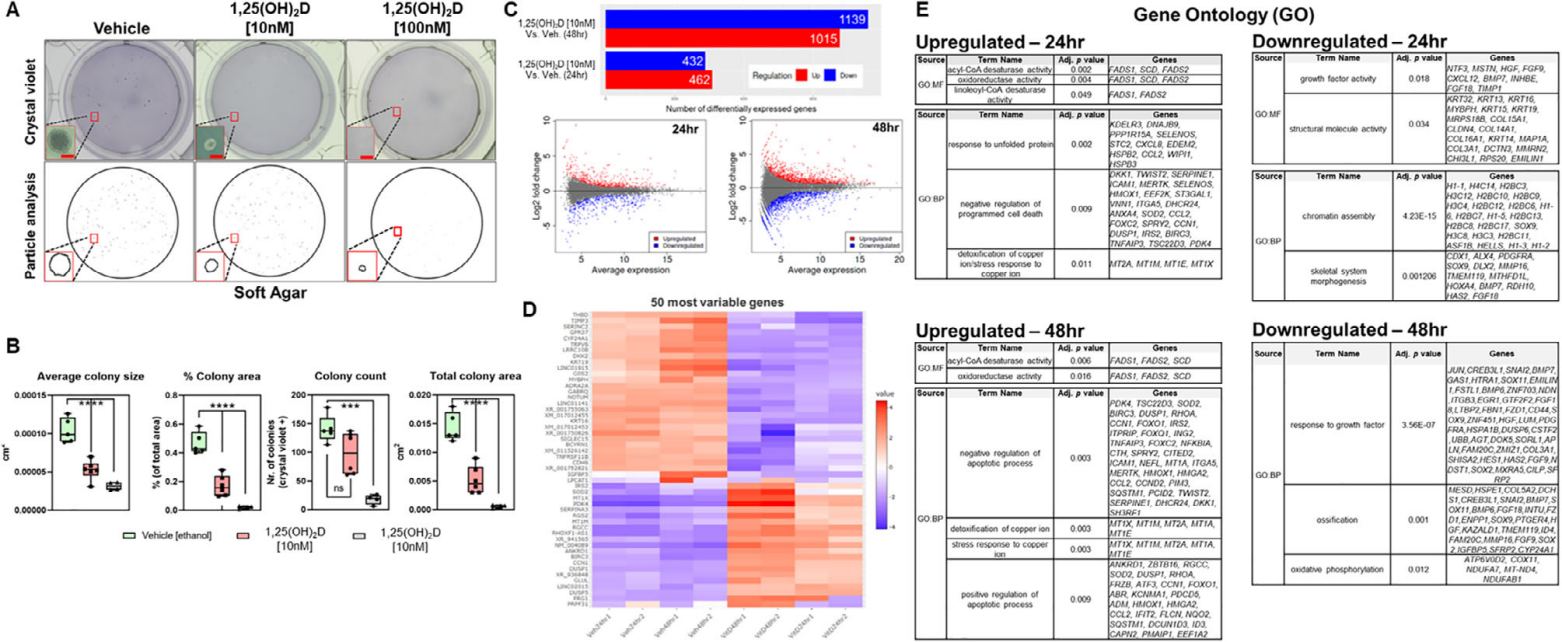
Genomewide assessment of 1,25(OH)_2_D‐mediated transcription using RNAseq. (*A*) Top: Representative macroscopic images of soft agar colony formation of MG‐63 cells treated with 1,25(OH)_2_D for 14 days. Bar = 100 μm. Bottom: ImageJ particle analysis of colonies. (*B*) Quantitation of the data from (*A*), summed from 5 to 6 representative macroscopic fields for each condition using data derived from ImageJ (*n* = 5–6). Data are presented as mean ± SEM error bars; ^****^ *p* ≤ 0.0001 and ^***^ *p* ≤ 0.001 (one‐way ANOVA with Tukey's multiple comparisons test). (*C*) MA plot and summary of differentially expressed genes (DEGs) based on DESeq2 method of RNAseq data. Plotted are the differences between measurements from 1,25(OH)_2_D [10 nM] versus vehicle‐treated cells by transforming the data onto M (log ratio) and A (mean average) scales. DEGs selected based on false discovery rate (FDR) set to 0.05 and log fold change^(^ ^1^ ^)^ compared with controls. (*D*) Heatmap with hierarchical clustering tree of the 50 most variable genes (*n* = 2 samples/condition). (*E*) Functional annotation and enrichment analysis using gene ontology (GO). Annotated genes, descriptors, and adjusted *p* values (≤0.05 considered significant) presented. GO molecular function (MF) and biological process (BP) domains are used to establish relationships. DEGs filtered using g:GOSt at https://biit.cs.ut.ee/gprofiler/gost.

Previous studies have shown that 1,25(OH)_2_D can suppress the growth of MG‐63 cells but not of receptor‐poor cell lines in standard 2D culture assays within the range of 100 nM (10^−8^ M or 40 ng/mL) and 10 nM (10^−9^ M or 4 ng/mL).^(^ ^26^ ^)^ However, 3D colony formation in soft agar is the gold‐standard assay to study anchorage‐independent carcinogenesis in vitro^(^ ^27^ ^)^ and has not been tested on 1,25(OH)_2_D treated MG‐63 cells to date. After 14 days of treatment, both 10 nM and 100 nM of 1,25(OH)_2_D resulted in a significant reduction in overall colony size (Fig. 1*A*, *B*). However, 10 nM of 1,25(OH)_2_D did not significantly affect the number of colonies after treatment, in contrast to 100 nM (Fig. 1*B*). This suggests that 1,25(OH)_2_D may induce cell apoptosis at the higher 100 nM concentration and that 1,25(OH)_2_D can suppress MG‐63 tumor growth at 10 nM via non‐apoptotic mechanisms and lower toxicity (see later).

To define the genomewide impact on transcription by 1,25(OH)_2_D on MG‐63 cells under the growth‐inhibiting conditions at 10 nM, high throughput RNAseq analysis was carried out. After 24 hours of continuous 1,25(OH)_2_D treatment, there were 462 and 432 differentially up‐ and downregulated genes, respectively, compared with vehicle treatment using a false discovery rate (FDR) threshold of 0.05 and log fold change (1) with a high degree of correlation (Pearson correlation coefficient = 0.96–0.99; principal component analysis) (Fig. 1 *C*, Supplemental Fig. S1, and Supplemental Worksheet S1). After 48 hours of 1,25(OH)_2_D treatment, there were 1015 and 1139 differentially up‐ and downregulated genes, respectively. To identify subgroups of genes that share expression patterns, we ranked genes by their standard deviation to obtain hierarchical clusters using DESeq2 (Fig. 1*D* and Supplemental Worksheet S2). An interactive heatmap of the 50 most variable genes shows that 1,25(OH)_2_D induced genes such as *SOD2*, *IRS2*, *BIRC3*, and *DUSP1/5*, which are either cytoplasmic or mitochondrial signaling molecules that mediate the effects of growth factors and/or cytokine interactions with known anticancer properties.^(^ ^28^ ^)^ For example, 1,25(OH)_2_D strongly induced the expression of the mitochondrial, but not cytosolic, manganese superoxide dismutase, SOD2, which converts the free radical O_2_^• −^ (superoxide) to H_2_O_2_ to defend against free radicals. The 50 most variable downregulated genes included the cytochrome P450 family 24 subfamily A member 1 (*CYP24A1*), the onco‐channel *TRPV6*, and *DKK2* (i.e., a Wnt mediator of tumor immune evasion). CYP24A1 functions as a mitochondrial monooxygenase that catalyzes the 24‐hydroxylation and catabolism of 1,25(OH)_2_D, suggesting a negative feedback response to preserve 1,25(OH)_2_D signaling and its anticancer effects in MG63 cells.

Next, we studied the relationships among the up‐ and downregulated genes relative to their enriched gene ontology (GO) terms using hierarchical clustering trees (Supplemental Fig. S2 *A*). The downregulated genes were overwhelmingly involved in nucleosome/chromatin assembly and organization, as well as DNA replication. Since nucleosomes assemble and become octameric during DNA replication amassing on daughter DNA strands, these findings suggest 1,25(OH)_2_D decreases replication, replication stress, and genomic instability associated with cancer. The decrease in nucleosome assembly also suggests that 1,25(OH)_2_D ‐treated MG‐63 cells may be in interphase of the cell cycle, supporting our previous studies,^(^ ^22^ ^)^ where DNA is less compact and associated with increased chromatin accessibility (see later). Although chromatin remodeling, per se, was not a feature of the analysis, we did identify chromatin‐modifying enzymes (e.g., SIRT1,4) that were differentially regulated after 1,25(OH)_2_D treatment. Nevertheless, the upregulated genes were associated with programmed cell death, translation, and response to organic substance. Of note, although regulators of apoptotic pathways were found to be enriched, we observed no changes in the early apoptosis marker Annexin V phosphatidylserine in 1,25(OH)_2_D‐treated MG‐63 cells at 10 nM (data not shown). We also used the dimension reduction algorithm, t‐SNE, to map the top genes, and then identified four clusters of enriched pathways called k‐means that were further mapped to GO biological processes (Supplemental Fig. S2 *B* and Supplemental Worksheet S3). Cluster A consisted of genes upregulated after 48 hours of 1,25(OH)_2_D treatment that was enriched for the defense response to virus pathway. Cluster B consisted of genes upregulated after 1,25(OH)_2_D treatment for both 24 and 48 hours that were enriched for the stress response pathway. Cluster C consisted of genes downregulated after 48‐hour 1,25(OH)_2_D treatment that enriched for the chromosome organization pathway. Lastly, Cluster D consisted of genes downregulated after both 24 and 48 hours that were enriched for chromatin/nucleosome assembly and cell development pathways. These findings show that 1,25(OH)_2_D regulates genome architecture and downstream stress response pathways as part of its anticancer response.

### 3.2 Functional enrichment analysis reveals 1,25(OH) _2_D‐mediated cancer inhibition via mitochondrial OXPHOS and stress regulators

Functional annotation and gene set enrichment analysis (GSEA) were performed using several methods to reflect the heterogeneity of data repositories and statistical approaches. We first used the g:GOSt program to map genes to known functional information to determine statistically significant enriched relationships. The data were stratified based on GO molecular functions (MF), biological processes (BP), and cellular components (Supplemental Worksheets S4 and S5). Based on GO‐MF subset analysis, genes that regulate fatty acid desaturases were upregulated after 1,25(OH)_2_D treatment, suggesting a putative role in unsaturated fatty acid biosynthesis and utilization (Fig. 1*E*). Based on GO‐BP, 1,25(OH)_2_D treatment induced genes that regulate unfolded proteins, programmed cell death, and the detoxification of metal ions. On the other hand, 1,25(OH)_2_D suppressed growth factors and structural molecule activity‐related genes based on GO‐MF. Based on GO‐BP, 1,25(OH)_2_D suppressed chromatin assembly, morphogenesis, and oxidative phosphorylation (OXPHOS)‐related genes. The OXPHOS genes include COX11, which is a copper‐binding subunit of the cytochrome c oxidase enzyme in the electron transfer chain in the mitochondria. Several respiratory chain NADH dehydrogenase subunit genes were also downregulated, including NDUFA7, MT‐ND4, and NDUFAB1. The remainder of the enriched pathways with large gene sets are included in Supplemental Worksheets S4 and S5, which also include genes enriched for telomere maintenance and adipogenesis, for example.

**Fig 2:**
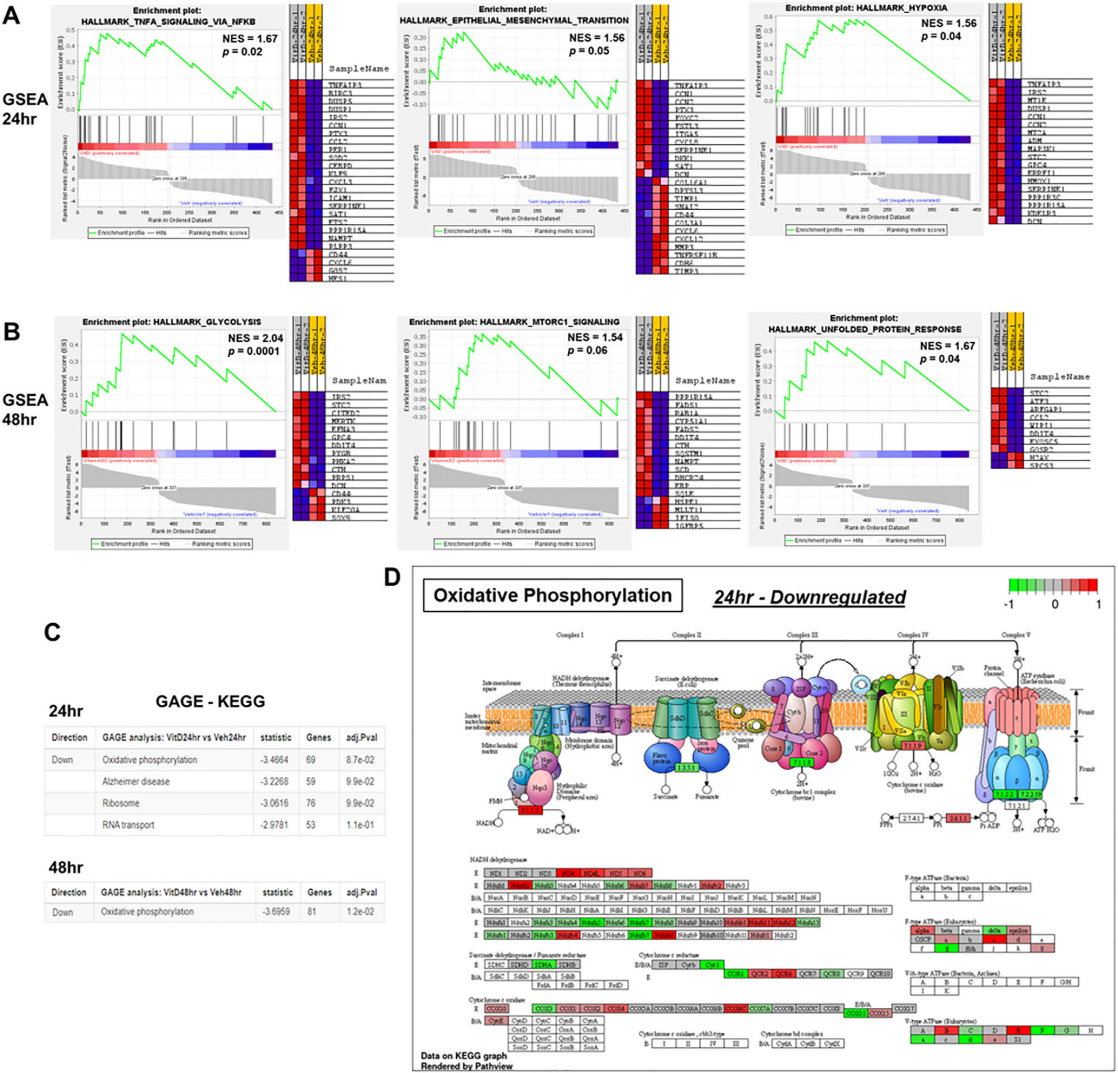
Gene Set Enrichment Analysis (GSEA) to identify pathways enriched in ranked gene lists after (*A*) 24 and (*B*) 48 hours of 1,25(OH)_2_D treatment. GSEA score curves depict the strength of gene sets in the Molecular Signature Database. “Signal‐to‐Noise” ratio (SNR) statistic was used to rank the genes per their correlation with either 1,25(OH)_2_D [10nM] treatment (red) or vehicle treatment (blue). Generally, the gene sets will be significant when a proportionally large number of genes fall in the upper or lower part of the distribution. The heatmap on the right of each panel depicts the genes contributing to the enriched pathway. The green curve corresponds to the enrichment score, which is the running sum of the weighted enrichment score obtained from GSEA‐positive normalized enrichment score (NES) and significant *p* values denote the most enriched pathways of the members of the gene set. Full pathway and gene list in Worksheet S7, GSEA performed at http://www.broad.mit.edu/gsea. (*C*) GAGE method for gene set enrichment of 1,25(OH)2Dtreatment. The fold change (log‐based) was used for the per gene statistics and adjusted p values (≤0.05 considered significant) presented. Full pathway and gene list in Worksheet S8, GAGE performed at https://pathview.uncc.edu/gageIndex. (*D*) Illustrative example of oxidative phosphorylation pathway found significantly enriched after 24 hours of 1,25(OH)2D treatment using Pathview Web (https://pathview.uncc.edu/). Differentially regulated gene list from 24 hours vehicle versus 1,25(OH)2D [10nM] comparison was inputted (i.e., the fold change (log‐based)).

We next performed GSEA in conjunction with the Molecular Signatures Database (MSigDB, version 7.3) of annotated gene sets. GSEA associates a treatment phenotype to a group or a list of weighted genes for comparison. The MSigDB gene sets are divided into nine major collections, whereby the hallmarks (H) gene sets (i.e., 50 gene sets) and canonical pathways (CP) gene sets (i.e., 189 gene sets) were applied with the cut‐off of *p* ≤ 0.05 to select biologically meaningful processes (Supplemental Worksheet S6). GSEA analysis of the 24‐hour 1,25(OH)_2_D‐treated samples revealed gene sets related to inflammation, hypoxia, and epithelial‐mesenchymal transition (EMT) pathways that were not discovered using g:GOSt (Fig. 2A). For example, the data suggest that 1,25(OH)_2_D can reverse EMT to suppress mesenchymal metastasis through downregulation of SNAI2, a key zinc finger transcription factor that maintains the loose mesenchymal phenotype (Fig. 2*A*, *B*). After 48 hours of 1,25(OH)_2_D treatment, the enriched pathways were related to hypoxia, glycolysis, inflammation, unfolded protein response, mTOR pathway, cholesterol homeostasis, apoptosis, xenobiotic metabolism, and p53 signaling (Fig. 2*B*). Key upregulated genes include DDIT4/REDD1 and sequestosome 1 (SQSTM1), which target the direct inhibition of mTOR or indirect effects through autophagy, respectively. In terms of hypoxia, decreased OXPHOS after 1,25(OH)_2_D treatment is likely to increase molecular oxygen levels as hypoxia in cancer cells is partly due to increasing O_2_ consumption and reduction to water that can thereby induce EMT.^(^ ^29^ ^)^ Hyperoxia is also supported by the increased SOD2 levels after 1,25(OH)_2_D treatment, as SOD2 metabolizes superoxide radicals into hydrogen peroxide. These findings suggest that 1,25(OH)_2_D affects major pathways involved in oxygen levels and the growth regulation of tumor cells.

In addition, we applied generally applicable gene‐set enrichment (GAGE) analysis that has no limitations on sample size based on a parametric gene randomization method to test the significance of gene sets using log‐based fold changes as the per gene statistic. By using the absolute values of fold change in the GAGE analysis combined with the Kyoto Encyclopedia of Genes and Genomes (KEGG) database, 1,25(OH)_2_D was shown to significantly downregulate a more dynamic OXPHOS gene set at both 24 and 48 hours of treatment (Fig. 2*C* and Supplemental Worksheet S7). The GAGE output was shared with Pathview to rationalize the OXPHOS genes (Fig. 2*D*), whereby the analysis shows that 1,25(OH)_2_D downregulates the second (II) and third (III) large enzyme complexes (i.e., succinate dehydrogenase and cytochrome bc1 complex, respectively) in the respiratory electron transport chain of MG‐63 cells. The cytochrome bc1 complex is responsible for the proton gradient as well as for the formation of O_2_^• −^. Disruption of the flow of electrons across the membrane is predicted to alter the transmembrane difference of proton electrochemical potential, which ATP synthases use to generate energy (see later). Furthermore, 1,25(OH)_2_D downregulated ATP synthase components (e.g., ATP5D, ATP5A1, ATP5C1) as another mode to regulate overall mitochondrial activity. Collectively, these findings suggest that 1,25(OH)_2_D promotes additional metabolic shifts that involve the suppression of mitochondrial OXPHOS as part of its anticancer strategy.

### 3.3 1,25(OH) _2_D‐mediated organellar hormesis enforces stress tolerance and growth inhibition of MG‐63 cells

**Fig 3:**
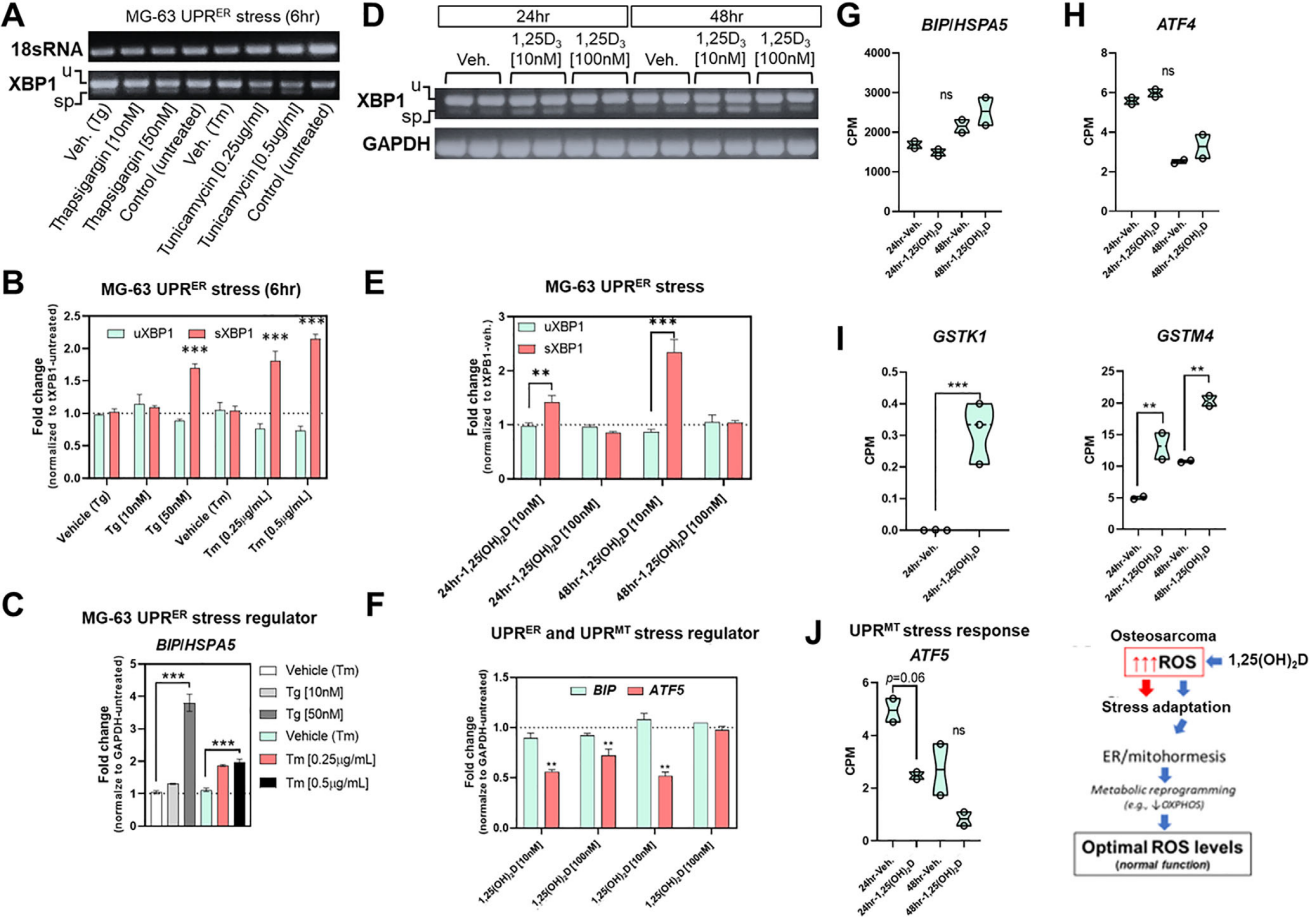
1,25(OH)_2_D and ER/mitochondrial unfolded protein stress regulation. (*A*) Representative endpoint PCR analysis of IRE1‐XBP1 expression after 6 hours of positive control treatments. u (unspliced XBP1; 256 bp), sp (spliced XBP1; 230 bp), 18sRNA (190 bp). (*B*) Real‐time PCR analysis of IRE1‐XBP1 expression after 6 hours of positive control treatments. The graph depicts fold change of either uXBP1 (unspliced) or sXBP1 (spliced) normalized to the total XBP1 levels. Data are presented as mean ± SEM error bars (*n* = 3 samples/condition); ^***^ *p* ≤ 0.001 (one‐way ANOVA with Tukey's multiple comparisons test compared with respective vehicle). (*C*) Real‐time PCR analysis of BIP/HSPA5 expression in positive controls. Data are presented as mean ± SEM error bars (*n* = 3 samples/condition); ^***^ *p* ≤ 0.001 (one‐way ANOVA with Tukey's multiple comparisons test compared with respective vehicle). (*D*) Representative endpoint PCR analysis of IRE1‐XBP1 expression after 24 to 48 hours of 1,25(OH)_2_D treatments. u (unspliced XBP1; 256 bp), sp (spliced XBP1; 230 bp), GAPDH (350 bp). (*E*) Real‐time PCR analysis of IRE1‐XBP1 expression after 24 to 48 hours of 1,25(OH)_2_D treatments. The graph depicts fold change of either uXBP1 (unspliced) or sXBP1 (spliced) normalized to the total XBP1 levels. Data are presented as mean ± SEM error bars (*n* = 3 samples/condition); ^***^ *p* ≤ 0.001, ^**^ *p* ≤ 0.01 (one‐way ANOVA with Tukey's multiple comparisons test compared with respective vehicle). (*F*) Real‐time PCR analysis of BIP/HSPA5 and ATF5 expression after 24 to 48 hours of 1,25(OH)_2_D treatments. Data are presented as mean ± SEM error bars (*n* = 3 samples/condition); ^**^ *p* ≤ 0.01 (one‐way ANOVA with Tukey's multiple comparisons test compared with respective vehicle). (*G–J*) RNAseq analysis of ER/mitochondrial stress and hormetic regulators. A two‐way ANOVA was performed with Bonferroni's multiple comparisons test using the counts per million (CPM) values (*n* = 2 samples/condition), where the *p* value summaries were depicted as ^****^ *p* ≤ 0.0001, ^***^ *p* ≤ 0.001, and ^**^ *p* ≤ 0.01. ns = not significant; UPR = unfolded protein response. (*K*) Proposed model: 1,25(OH)_2_D enforces stress tolerance in cancer cells via metabolic reprogramming involving ER/mitohormesis.

Our genomewide bioinformatics analysis suggests the involvement of the unfolded protein response (UPR) in 1,25(OH)_2_D‐treated MG‐63 cells, which is known to mediate stress tolerance and organismal longevity involving a process called hormesis.^(^ ^30^ ^)^ Hormesis describes a phenomenon where mild cellular stress caused by unfolded proteins stimulates alternative signaling pathways with beneficial, overcompensating outcomes and organellar connectivity.^(^ ^30^ ^)^ To better understand how 1,25(OH)_2_D modulates hormetic responses in cancer cells, we investigated various ER and mitochondrial hormetic signaling pathways that involve antioxidants and protein‐folding chaperones (Fig. 3). The ER transmembrane receptor protein kinases (ER‐TRK) IRE1 and PERK and the transcription factor ATF6 govern the expression of factors that protect cells as part of the hormetic UPR by promoting cell cycle arrest, protein translation inhibition, and chaperone production. A proxy for activated IRE1 is cleavage of 26 base pairs from its substrate, XBP1, to generate a spliced form called sXBP1 that functions as a transcription factor for expression of binding immunoglobulin protein (BIP, also called GRP78 or HSPA5), which functions as a major ER stress chaperone. To characterize UPR in the MG‐63 cell system, thapsigargin and tunicamycin (i.e., blockers of the ER ATPase/SERCA pump and glycoprotein synthesis, respectively) were first used and found to induce a dose‐dependent increase in sXBP1 and BIP/HSPA5 (Fig. 3*A–C*). Interestingly, 1,25(OH)_2_D treatment enhanced sXBP1 in a time‐dependent manner at 10 nM but not at 100 nM (Fig. 3*D*, *E*) with no change in BIP mRNA levels across all concentrations, suggesting a hormetic response to insoluble proteins (Fig. 3*G*, *H*). As the proxies for ATF6 activation are upregulation of BIP and uXBP1, our findings also suggest that ATF6 plays a minimal role in the 1,25(OH)_2_D response (Fig. 3*E*). Two proxies for PERK activation are ATF4 and CHOP (also called DDIT3 or GADD153), whereby RNAseq analysis showed no changes in both transcripts after 1,25(OH)_2_D treatment (Fig. 3*H* and Supplemental Worksheet S1). Therefore, we investigated potential hormetic antioxidative responses of the alternative ER‐TRK, recently described in *C. elegans*,^(^ ^31^ ^)^ in the context of 1,25(OH)_2_D by appraising the human glutathione S‐transferase family of genes. We only observed statistically significant increases in glutathione S‐transferase kappa 1 (GSTK1) and glutathione S‐transferase Mu 4 (GSTM4) after 1,25(OH)_2_D treatment of MG‐63 cells (Fig. 3*I*), whereby lower levels of GSTK1 have been linked to the elevation of mt ROS underlying hypertrophic cardiomyopathy.^(^ ^32^ ^)^ Lastly, since the bioinformatics analysis also suggests the downregulation of OXPHOS, we assessed mitochondrial UPR by way of activating transcription factor 5 (ATF5) (Fig. 3*J*). ATF5 is a major mitochondrial stress regulator that can induce proteostasis and chaperonin production,^(^ ^33^ ^)^ whereby 10 nM of 1,25(OH)_2_D treatment significantly downregulated ATF5 in MG‐63 cells, the effect of which dissipated at higher concentrations, signifying a hormetic response (Fig. 3*J*). Overall, the results suggest that 1,25(OH)_2_D activates distinct hormetic adaptive responses in the ER and mitochondria to regain control of the growth of cancer cells, which may underly beneficial interorganellar communication to overcome cancer stress (Fig. 3*K*).

### 3.4 A multi‐omics approach to study mitochondrial anticancer responses to 1,25(OH) _2_D

**Fig 4:**
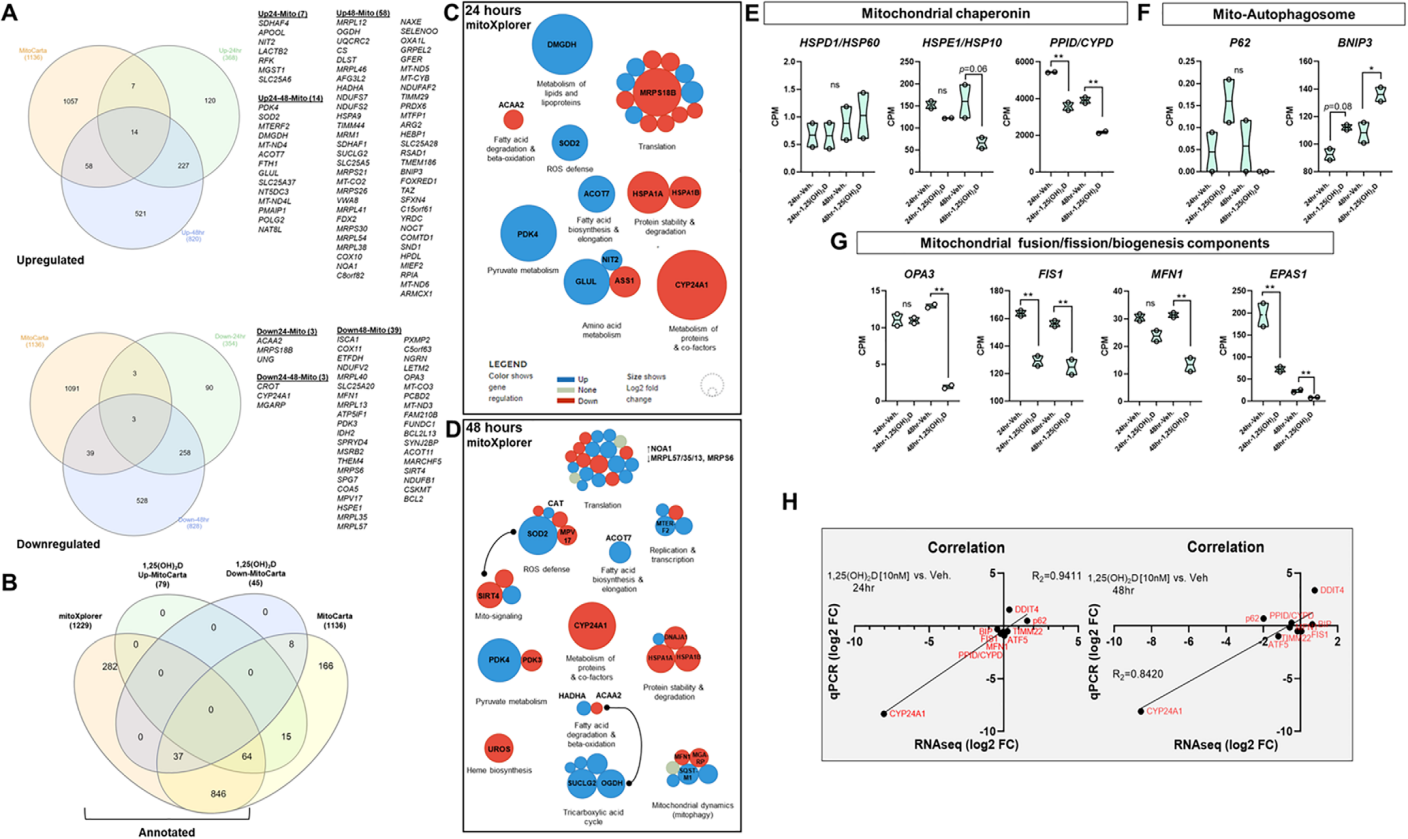
A multi‐omics approach to study mitochondrial anticancer responses to 1,25(OH)_2_D. (*A*) Identification of mitochondria‐related genes from 1,25(OH)_2_D treated MG‐63 cells using MitoCarta. Differentially expressed genes (DEGs) from both the 24‐ and 48‐hour data sets were cross‐referenced to the MitoCarta database. Venn analysis was performed at http://www.interactivenn.net. (*B*) Identification of annotated 1,25(OH)_2_D‐mediated mitochondrial genes. Mitochondrial DEGs derived from MitoCarta were cross‐referenced with the annotated database mitoXplorer. Venn analysis was performed at http://www.interactivenn.net. (*C, D*) Mitochondrial interactome of 1,25(OH)_2_D treated MG‐63 cells. Functional relationships were characterized by mitochondrial DEGs using the mitoXplorer software. http://mitoxplorer.ibdm.univ‐mrs.fr/. (*E–G*) RNAseq analysis of mitochondrial stress, biogenesis, and clearance regulators. A two‐way ANOVA was performed with Bonferroni's multiple comparisons test using the counts per million (CPM) values (*n* = 2 samples/condition), where the *p* value summaries were depicted as ^**^ *p* ≤ 0.01 and ^*^ *p* ≤ 0.05. ns = not significant. (*H*) Real‐time PCR validation of select RNAseq data. Plots showing correlation (R_2_ = 0.94–0.84) between sample sets (*n* = 3 samples/condition).

Given that 1,25(OH)_2_D suppresses mitochondrial UPR, we performed a more granular multi‐omics assessment of mitochondrial transcriptional changes using the annotated databases MitoCarta and mitoXplorer. MitoCarta currently annotates 1136 genes encoding mitochondrial proteins, while mitoXplorer contains 1229 genes. First, we used MitoCarta (version 3.0) to identify differentially regulated mitochondria‐related genes from our RNAseq data set.^(^ ^34^ ^)^ Among the 1477 upregulated 1,25(OH)_2_D‐mediated differentially expressed genes (DEGs) (Fig. 1), we identified 79 genes that encode mitochondria proteins within the combined 24‐ and 48‐hour gene sets (~5%; Fig. 4*A* and Supplemental Worksheet S8). Among the 1571 downregulated 1,25(OH)_2_D‐mediated DEGs (Fig. 1), we identified 45 genes encoding mitochondrial proteins in total (~2.8%; Fig. 4*A* and Supplemental Worksheet S8). However, MitoCarta provides no annotation on the genes, and to understand the biological significance behind these changes, we utilized the annotated mitoXplorer (version 1.0) necessary for pathway analysis. In all, there were 64 and 37 1,25(OH)_2_D‐mediated up‐ and downregulated mitochondrial genes, respectively, that were common between the two repositories (Fig. 4*B*). There were only 15 and 8 up‐ and downregulated 1,25(OH)_2_D ‐mediated mitochondrial genes, respectively, that were specific to the MitoCarta repository and not included in the mitoXplorer annotative analysis. Based on the mitoXplorer analysis, the 1,25(OH)_2_D‐mediated downregulated DEGs after 24 hours included *MRPS18B*, which encodes a 28S subunit mitoribosomal protein involved in protein translation (Fig. 4*C* and Supplemental Worksheet S8). In addition, HSPA1A and B, members of the heat shock protein family A were also downregulated by 1,25(OH)_2_D, suggesting a lowering of stress aggregation and increased protein stability in mitochondria. In terms of metabolism, dimethylglycine dehydrogenase (DMGDH), a mitochondrial enzyme involved in phosphatidylcholine and lipid metabolism and glycine modifications, was elevated after 1,25(OH)_2_D treatment (Fig. 1*E*). Recently, studies have shown that DMGDH can play a role in antioxidant defense,^(^ ^35^ ^)^ and low levels can be a diagnostic and prognostic marker for hepatocellular carcinoma metastasis by acting on the Akt pathway.^(^ ^36^ ^)^ Genes that regulated beta oxidation of fatty acids were also found to be suppressed by 1,25(OH)_2_D treatment (↓ACAA2), suggesting another mean for ROS reduction.^(^ ^37^ ^)^ Interestingly, mitochondrial amino acid metabolism and detoxification were upregulated after 1,25(OH)_2_D treatment by way of glutamate‐ammonia ligase (GLUL), which is a mitochondrial enzyme that catalyzes the synthesis of glutamine from the more toxic glutamate and ammonia. In addition, nitrilase omega‐amidase (NIT2) was upregulated by 1,25(OH)_2_D, which is known to play a role in arresting cells to remove toxic intermediates such as 2‐oxoglutaramate.^(^ ^38^ ^)^ Pyruvate metabolism was also affected after 1,25(OH)_2_D treatment by way of upregulation of the mitochondrial pyruvate dehydrogenase kinase 4 (PDK4). PDK4 inhibits the mitochondrial pyruvate dehydrogenase complex to reduce pyruvate conversion from glucose, suggesting that 1,25(OH)_2_D may conserve glucose metabolism (i.e., slowing glycolysis), as during hibernation, by decreasing its conversion to acetyl‐CoA.

In the 48‐hour analysis, the overwhelming effect of 1,25(OH)_2_D on mitochondrial protein translation at 24 hours was aborted, suggesting adaptive responses (Fig. 4*D*). Additional selective pressures toward translation occurred via upregulation of MTERF2, a transcription termination factor that modulates cell growth and the cell cycle.^(^ ^39^ ^)^ Longer treatments of 1,25(OH)_2_D did enhance the ROS defense response (↑CAT); however, this was countered by decreased MPV17, which is involved in ROS neutralization and mitochondrial protection.^(^ ^40^ ^)^ Antioxidant responses closely regulate mitochondrial epigenetic signaling factors such as SIRT4,^(^ ^41^ ^)^ an enzyme with deacetylase and ADP‐ribosylation activities, which was downregulated after 1,25(OH)_2_D treatment, suggesting a mode for further fine‐tuning of epigenomic regulation. Other mitochondrial metabolic and dynamic effects of 1,25(OH)_2_D include the suppression of the heme biosynthesis pathway by way of UROS, which is part of the catalytic steps of porphyrin biosynthesis and associated with cancer when heme production is left unchecked.^(^ ^42^ ^)^ Furthermore, mitofusion 1 (MFN1) was downregulated after 1,25(OH)_2_D treatment that mediates mitochondrial fusion, suggesting reduced mitochondrial networks, ATP production, and OXPHOS. SQSTM1, a protein involved in mitophagy, was upregulated after 1,25(OH)_2_D treatment, suggesting a selective and adaptive process to remove dysfunctional mitochondria from cancer cells. The TCA cycle, which provides electrons via the reducing agent NADH for OXPHOS, was enhanced after 48 hours of 1,25(OH)_2_D treatment despite the suppression of OXPHOS, raising the possibility of non‐redox roles.^(^ ^43^ ^)^ For example, 1,25(OH)_2_D may involve substrate‐level phosphorylation as a metabolic reaction to generate energy instead of OXPHOS. SUCLG2, a GTP‐specific beta subunit of succinyl‐CoA synthase that forms succinyl‐CoA, succinate, and ATP through the coupling of this reaction independent of OXPHOS, was elevated after 1,25(OH)_2_D treatment. Also, OGDH, a dehydrogenase that catalyzes the conversion of 2‐oxoglutarate to succinyl‐CoA and carbon dioxide, was increased after 1,25(OH)_2_D treatment, which may further drive energy production via TCA non‐redox intermediates.

Lastly, several known mitochondrial genes were not co‐curated in the MitoCarta and mitoXplorer repositories, including DDIT4/REDD1^(^ ^44^ ^)^ (see later) that were validated by qPCR derived from our RNAseq data sets (Fig. 4*E–H*). We also observed a consistent downregulation of known mitochondrial chaperonin PPID (cyclophilin D). Although there was an upward trend for the mitophagy marker, P62 (Fig. 4*F*), qPCR reanalysis showed a statistically significant increase in transcript levels (Fig. 4*H*), suggesting a possible role in conjunction with SQSTM1 toward mitophagy. In addition, mitochondrial BCL2/adenovirus E1B 19 kDa protein‐interacting protein 3 (BNIP3) transcripts were increased after 1,25(OH)_2_D treatment and may interact with LC3 to remove damaged ER and mitochondria to recycle cellular content to promote the health of cells (Fig. 4*F*). Again, in terms of mitochondrial dynamics, 1,25(OH)_2_D treatment resulted in the downregulation of mitochondrial fission transcript, FIS1, and of OPA3, a dynamin‐related GTPase that regulates the equilibrium between mitochondrial fusion and mitochondrial fission (Fig. 4*G*). Endothelial PAS domain protein 1 (EPAS1) mRNA, which encodes a protein involved in mitochondrial biogenesis, was decreased after 1,25(OH)_2_D treatment (Fig. 4*G*). Overall, the multi‐omics approach revealed novel factors and pathways as part of 1,25(OH)_2_D's mitochondrial‐mediated anticancer response.

### 3.5 1,25(OH) _2_D‐mediated epigenetic regulation of mitochondrial‐related genes in MG‐63 osteosarcoma cells

**Fig 5:**
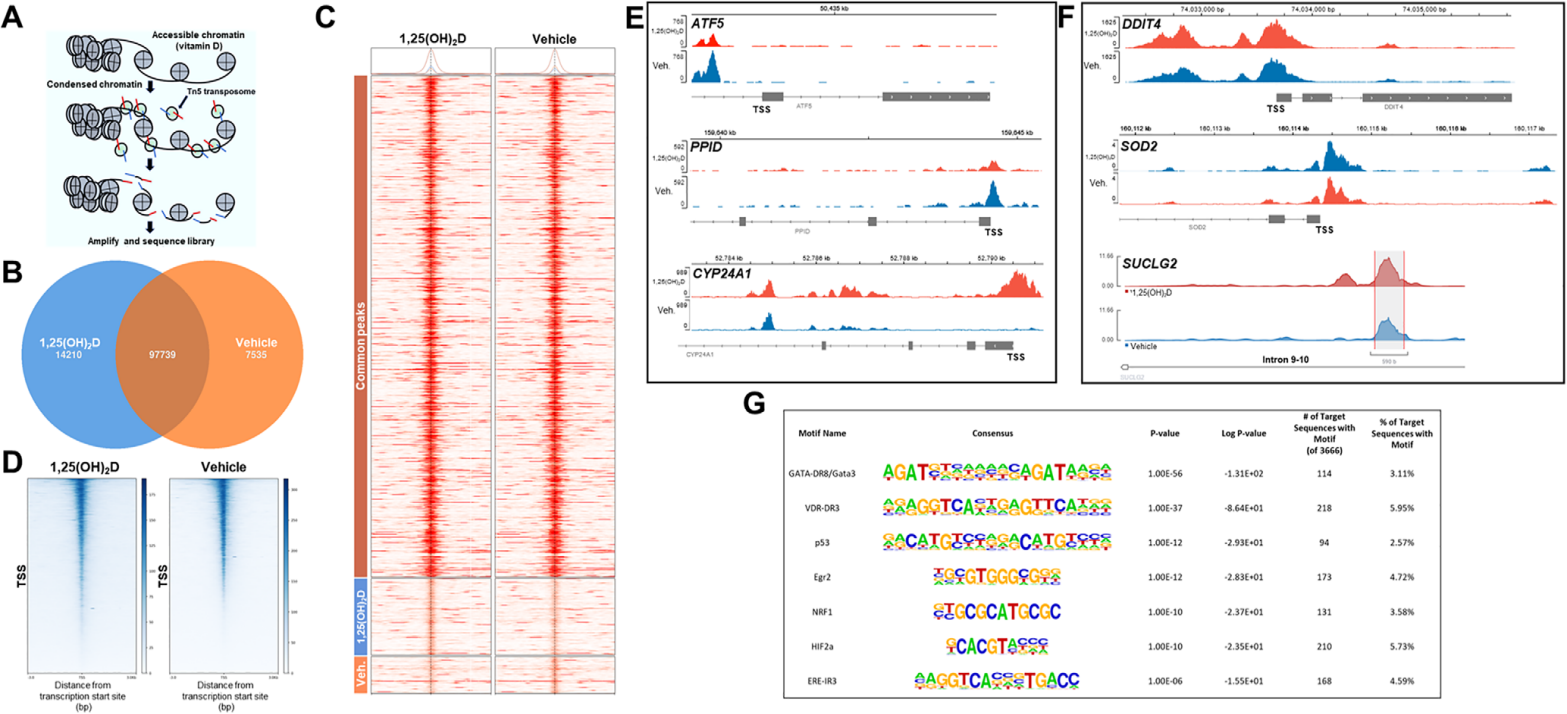
VDR‐mediated epigenetic regulation of MG‐63 osteosarcoma cells. (*A*) Changes in chromatin accessibility assayed by ATACseq. ATACseq identifies regions of open chromatin using Tn5 transposases and tagging sites with sequencing adaptors. (*B*) Global peak overlaps and peaks unique to 1,25(OH)_2_D [10 nM] and vehicle treatments for 24 hours. (*C*) Heatmap depicting the pattern of peaks outlined in the Venn diagram. There is one column per group used in the comparison. The color bars on the left correspond to Venn diagram grouping of peaks. The heatmap displays the read coverage density (redder means more reads at that location), with each row corresponding to the average peak profile for a single peak that is averaged within each group, across all groups in the comparison. (*D*) Heatmap for transcriptional start sites (TSS) after 1,25(OH)_2_D [10 nM] and vehicle treatments. Chromatin accessibility at TSS significantly increased in response to 1,25(OH)_2_D. The lower half of the 1,25(OH)_2_D plot harbors the 1,25(OH)_2_D‐unique peaks from (*B*) and (*C*). The plots specify a window of ± 3000 bp around the TSS of genes. Read density scores presented as the right index. (*E*) Genome browser track of ATACseq results for select mitochondrial downregulated genes. Differentially accessible regions identified for select genes. (*F*) Genome browser track of ATACseq results for select mitochondrial upregulated genes. Of note, a 1,25(OH)_2_D‐unique peak was identified in intron 9–10 of *SUCLG2*. (*G*) List of transcription factor (TF) motifs enriched in accessible chromatin upon 1,25(OH)_2_D treatment.

Next, to identify functional chromosomal regions that may govern anticancer responses and may be coregulated by 1,25(OH)_2_D and oxidative stress, we used assay for transposase‐accessible chromatin using sequencing (ATACseq) and assessment of transcription factor (TF) binding motifs. This method appraises genomewide chromatin accessibility using hyperactive Tn5 transposase that inserts sequencing adapters into open chromatin regions (Fig. 5*A*). The data show that most peaks were located within intronic, intergenic, and promoter regions across samples with 96% to 97% of reads with ≥Q30 scores, satisfying the quality‐control requirements (Supplemental Worksheet S9). Globally, there were 97,739 overlapping peaks, 14,210 1,25(OH)_2_D unique peaks, and 7535 vehicle‐unique peaks after 1,25(OH)_2_D treatment for 24 hours (Fig. 5*B*, *C*). The ATACseq results confirmed the RNAseq analysis showing an increased number of transcriptional start sites (TSS) that contain 1,25(OH)_2_D unique peaks at the expense of decreased nucleosome assembly (Fig. 5 *D* and Supplemental Fig. S2). Because many *cis*‐regulatory elements are close to the TSS of their targets, the data suggest that 1,25(OH)_2_D promotes global chromatin accessibility, enrichment, and transcriptional regulation from the TSS. To get further insight, we compared key down‐ and upregulated mitochondria‐related transcripts identified with RNAseq to the list of significant peaks. For downregulated genes (Fig. 5*E*), 1,25(OH)_2_D treatment resulted in decreased chromatin accessibility at the distal promoter region of *ATF5*, suggesting possible regulation by negative 1,25(OH)_2_D response elements.^(^ ^45^ ^)^ *PPID* gene expression is directly regulated by ATF5, and we observed a similar decrease in chromatin accessibility at the TSS and proximal protomer region. Interestingly, *CYP24A1* was one of the most downregulated genes identified after 1,25(OH)_2_D treatment, yet exhibited enhanced chromatin accessibility at both the TSS, proximal promoter, and as well as within intron 3–4, suggesting the possible “looping” of chromosomal structures that may suppress and discriminate CYP24A1 transcription in *trans* after 1,25(OH)_2_D treatment.^(^ ^46^ ^)^ For upregulated genes (Fig. 5*F*), we observed enhanced chromatin accessibility at both the proximal and promoter regions of *DDIT4*. On the contrary, there appeared to be nominal epigenetic regulation of *SOD2* by 1,25(OH)_2_D. This finding suggests either posttranslational and/or posttranscriptional modes of SOD2 mRNA regulation after 1,25(OH)_2_D treatment. Interestingly, one of the most significantly affected chromosomal regions identified by ATACseq was in intron 9–10 of the *SUCLG2* gene, suggesting a regulatory epigenetic mechanism induced by 1,25(OH)_2_D to control succinyl‐CoA synthase expression.

We next used hypergeometric optimization of motif enrichment (HOMER) for transcription factor (TF) motif discovery within 1,25(OH)_2_D‐sensitive open chromatin (Supplemental Worksheet S10).^(^ ^47^ ^)^ From this analysis, GATA3 was the most highly associated TF identified (Fig. 5*G*), whereby GATA3 is essential for normal tissue development^(^ ^48^ ^)^ and is commonly mutated in breast cancers.^(^ ^49^ ^)^ Not surprisingly, the VDR motif was the second most highly correlated nuclear TF identified from our analysis. Interestingly, we also identified the nuclear respiratory factor 1 (NRF1), a redox‐sensitive member of the Cap‐N‐Collar family of TFs that binds to antioxidant response elements (AREs),^(^ ^50^ ^)^ as a potential regulator of 1,25(OH)_2_D‐mediated epigenetic responses, suggesting that AREs may cooperate with 1,25(OH)_2_D response elements (VDREs) via NRF1‐VDR binding. 1,25(OH)_2_D treatment also may promote estrogen receptor binding, suggesting a synergistic effect to help promote the normal bone‐forming osteoblast phenotype in osteosarcoma cells.^(^ ^51^ ^)^ Overall, 1,25(OH)_2_D promotes chromatin accessibility in MG‐63 cancer cells to enhance the regulatory effects of specific TFs that may play important roles in oxidative stress defense and normal tissue and cellular developmental processes.

### 3.6 1,25(OH) _2_D‐mediated decrease in ΔΨ_M_ inhibits mitochondrial ROS production

The transcriptomic and epigenomic data thus far suggest that 1,25(OH)_2_D regulates mitochondrial functions in MG‐63 cells to promote its anticancer effects. Therefore, we investigated the mitochondrial membrane potential (ΔΨ_M_) using the ratiometric JC‐1 dye, where the accumulation of cationic J‐aggregates (red) in mitochondrial membranes acts as a proxy for polarized mitochondria. On the other hand, cells that have diminished ΔΨ_M_ will contain JC‐1 in its monomeric form (green) in either the mitochondria or cytoplasm during transition states.

**Fig 6:**
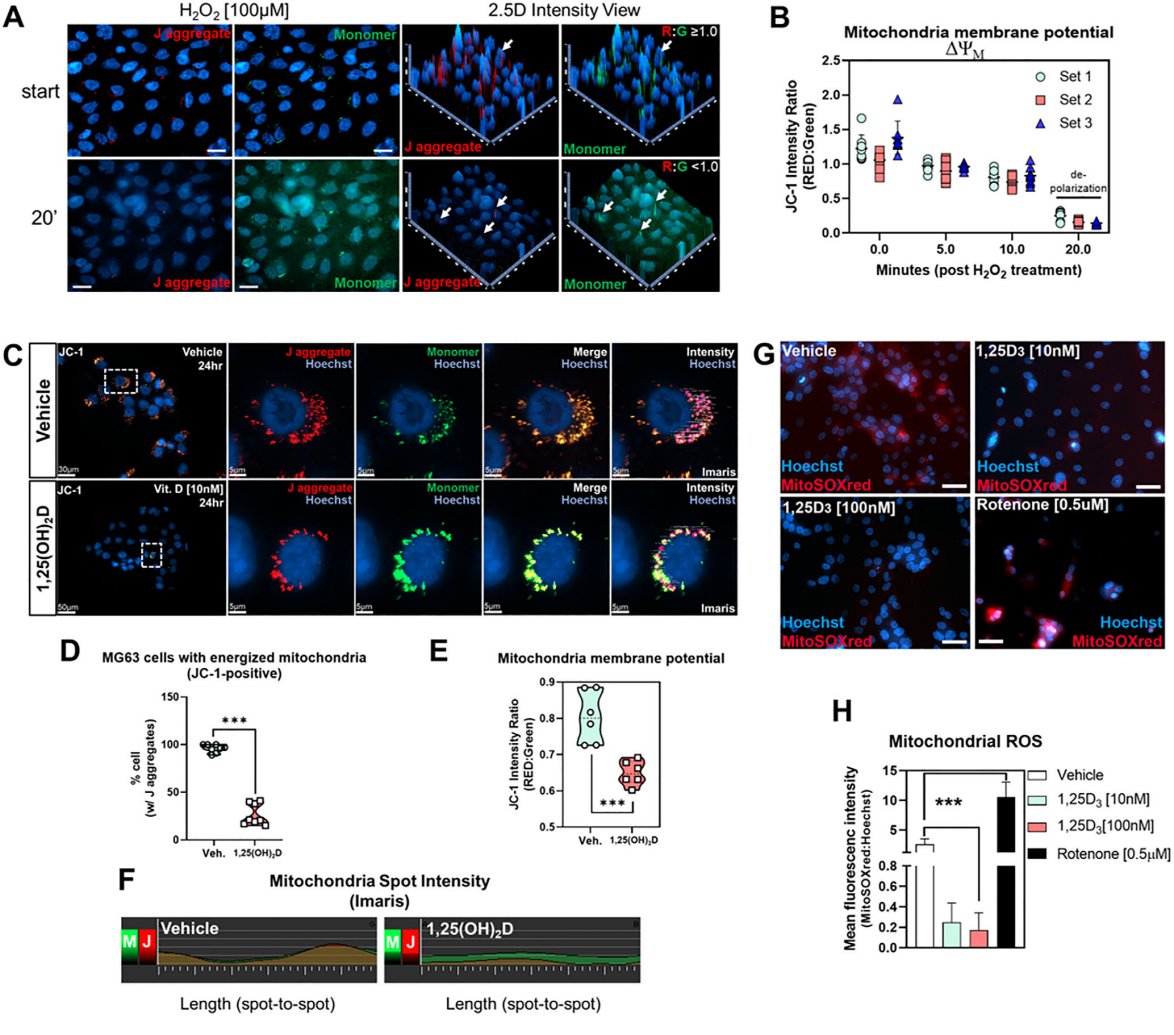
1,25(OH)_2_D depolarizes the mitochondrial membrane and inhibits ROS production. (*A*) Cytofluorimetric evaluation of the mitochondria membrane potential (ΔΨ_M_) using the dye JC‐1. High levels of hydrogen peroxide (H_2_O_2_) depolarized the mitochondria of MG‐63 cells over time. In the 2.5D view, intensity values in a two‐dimensional image were converted into a height map. The highest‐intensity values are represented by the greatest extension in the Z‐direction depicted by arrows. Simultaneous J aggregate (red) and monomeric JC‐1 dye (green) were measured at start and up to 20 minutes later. (*B*) Quantification of ΔΨ_M_ after H_2_O_2_ treatment. (*C*) ΔΨ_M_ measured using the JC‐1 cationic dye in MG‐63 cells. (*D*) Quantification of MG‐63 cells with J aggregates after 1,25(OH)_2_D [10 nM] and vehicle treatments for 24 hours. Data are presented as mean ± SEM error bars (*n* = 8 replicates/condition); ^***^ *p* ≤ 0.001 (two‐way ANOVA with Sidak's multiple comparisons test compared with vehicle). (*E*) Quantification of JC‐1 intensity ratio in MG‐63 cells treated with 1,25(OH)_2_D [10 nM] and vehicle for 24 hours. Data are presented as mean ± SEM error bars (*n* = 6 replicates/condition); ^***^ *p* ≤ 0.001 (two‐way ANOVA with Sidak's multiple comparisons test compared with vehicle). (*F*) J aggregate and monomer dynamics in MG‐63 cells. The spot intensity tool (Imaris) was used to determine the intensity (*y* axis) and position (*x* axis) between spot‐to‐spot (i.e., a mitochondria‐to‐mitochondria series) in treated cells. M = monomer; J = J aggregate. (*G*) Mitochondrial superoxide detection in MG‐63 cells after 1,25(OH)_2_D [10 nM] and vehicle treatments for 24 hours. Bar = 20 μm. (*H*) Quantification of mitochondrial superoxide in MG‐63 cells treated with 1,25(OH)_2_D [10 nM] and vehicle for 24 hours. Data are presented as mean ± SEM error bars (*n* = 6 replicates/condition); ^***^ *p* ≤ 0.001 (one‐way ANOVA with Tukey's multiple comparisons test compared with vehicle).

To validate the JC‐1 dye, we treated MG‐63 cells with hydrogen peroxide (H_2_O_2_), a known oxidant and mitochondrial membrane depolarizer.^(^ ^52^ ^)^ Within 20 sections of H_2_O_2_ treatment, we observed a decrease in the J‐aggregate‐to‐monomer ratio signifying a decrease in ΔΨ_M._ (Fig. 6*A*, *B*). In the 1,25(OH)_2_D studies, we pretreated MG‐63 cells for 24 hours and then measured the JC‐1 intensity ratios (Fig. 6*C*). Interestingly, while most of the vehicle‐treated MG‐63 cells were positive for J‐aggregates, only ~25% of 1,25(OH)_2_D‐treated cells contained J‐aggregates in their mitochondria, suggesting a complete collapse of the ΔΨ_M_ within most cells (Fig. 6*D*). Among those 1,25(OH)_2_D‐treated cells that exhibited J‐aggregates, their JC‐1 intensity ratio was significantly reduced compared with vehicle treatment (Fig. 6*C*, *E*). Using the Imaris software (Bitplane) spot intensity tool, the vehicle‐treated cells exhibited an overlap in J‐aggregate‐to‐monomer signals across a series of mitochondria (i.e., spot‐to‐spot) within cells (Fig. 6*F*). On the contrary, 1,25(OH)_2_D‐treated cells exhibited an increased level of non‐overlapping monomer‐to‐J‐aggregate signals, suggesting the depolarization of the mitochondria membrane and extramitochondrial presence of the monomers.

A key factor in determining the fate of cells with depolarized mitochondria is the level of ROS. To determine the impact of 1,25(OH)_2_D on ROS production within MG‐63 cells, we measured mitochondria‐specific ROS using the MitoSOX Red, a mitochondrial O_2_^• −^ indicator for live cells (Fig. 6*G*). 1,25(OH)_2_D treatment for 24 hours significantly reduced the production of O_2_^• −^ within MG‐63 cells compared with vehicle‐treated samples (Fig. 6*H*). Conversely, treatment with the inhibitor of complex I of the respiratory chain, rotenone, significantly increased mt ROS levels. Thus, MG‐63 osteosarcomas are accompanied by mechanisms that prevent mitochondrial depolarization, resulting in chronic intracellular mt ROS. Overall, the data suggest that 1,25(OH)_2_D treatment is associated with the opening of the mitochondrial permeability pores, loss of the electrochemical proton gradient, and reduced ROS as part of its anticancer effects.

### 3.7 1,25(OH) _2_D modulates mitochondrial structure and dynamics in MG‐63 cancer cells

**Fig 7:**
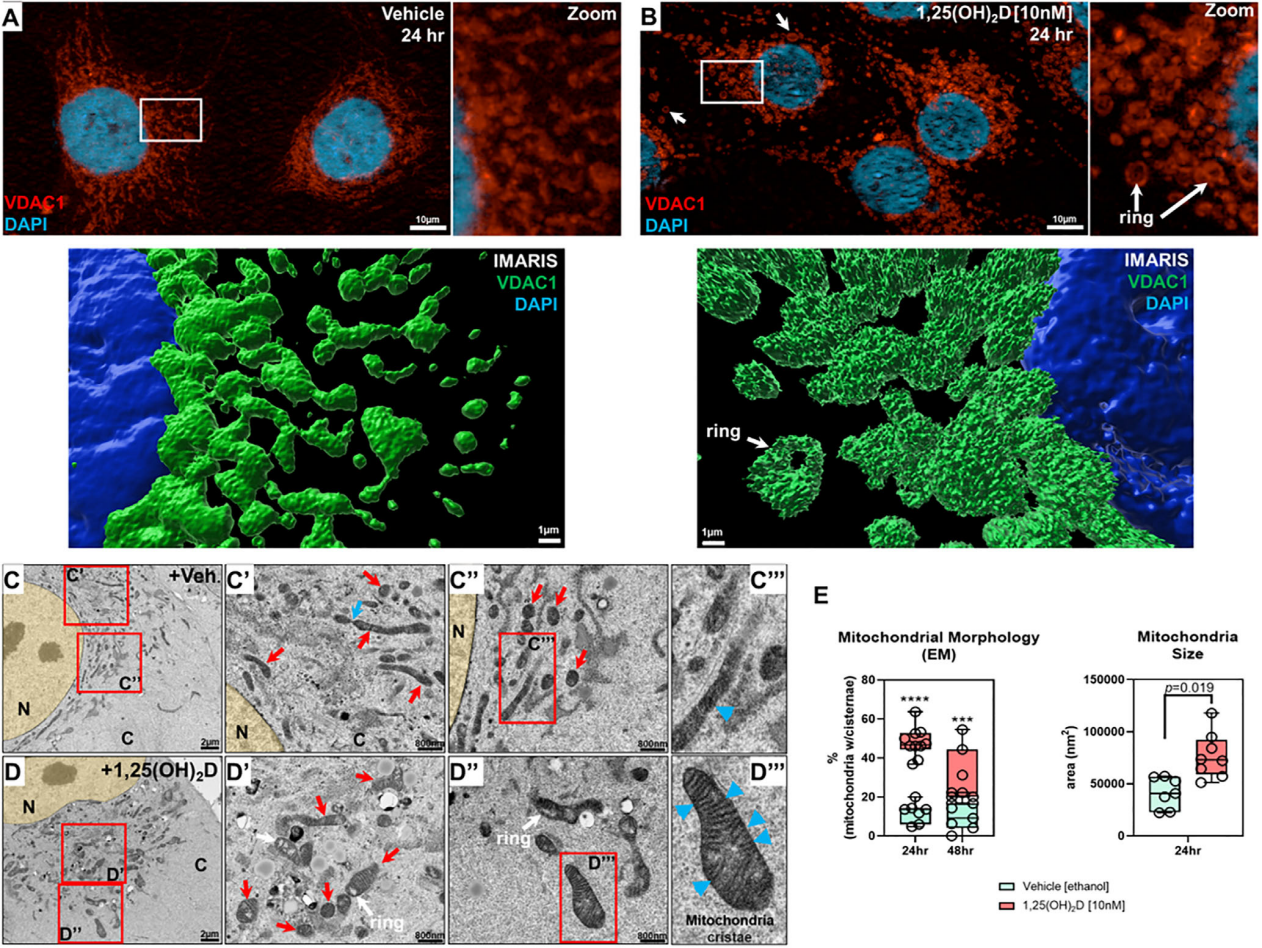
1,25(OH)_2_D modulates mitochondria structure in MG‐63 osteosarcoma cells. (*A*) Immunofluorescence labeling of VDAC1 within vehicle‐treated MG‐63 cells. Right panel is the magnification of the inset. The lower panel is Imaris 3D‐rendered image of the inset. (*B*) Immunofluorescence labeling of VDAC1 within 1,25(OH)_2_D [10 nM] treated MG‐63 cells. The right panel is the magnification of the inset. Arrows depict mitochondrial ring‐like structures. The lower panel is Imaris 3D‐rendered image of the inset. (*C*, *C′*, *C″*, *C′′′*) Representative transmission electron microscopy (TEM) images of vehicle‐treated MG‐63 cells for 24 hours. Red insets are marked with panel identifiers. (*C′*) Blue arrow depicts tethered mitochondria, and red arrows depict tubular mitochondria. (*C″*) Red arrows depict electron‐dense cross sections of tubular mitochondria. (*C′′′*) Blue arrowhead depicts loosely structured cristae. C = cytoplasm; N = nucleus. (*D*, *D′*, *D″*, *D′′′*) Representative TEM images of 1,25(OH)_2_D [10 nM] treated MG‐63 cells for 24 hours. Red insets are marked with panel identifiers. (*D′*) Red arrows depict mitochondria in various stages, e.g., tubular, herniated, swollen, with visible cristae. White arrows depict rings in mitochondria. (*D′′′*) Blue arrowheads depict defined cristae structures in mitochondria. (*E*) Quantification of TEM. For analysis, 7 to 10 cells were investigated per condition, in which we averaged parameters from 20 to 40 mitochondria per cell. Data are presented as mean ± SEM error bars (*n* = 7–10 cells/condition); ^****^ *p* ≤ 0.0001, ^***^ *p* ≤ 0.001 (two‐way ANOVA with Bonferroni's multiple comparisons test compared with vehicle).

We next appraised mitochondria structure and morphology using immunofluorescence (IF) and electron microscopy (EM). Using antibodies against the outer mitochondrial membrane voltage‐dependent anion‐selective channel 1 (VDAC‐1), we observed the classic elongated tubular shape of mitochondrial in vehicle‐treated MG‐63 cells (Fig. 7*A*). However, 1,25(OH)_2_D treatment promoted changes from a tubular to globular “ring” morphology with weak fluorescence in the center of mitochondria (Fig. 7*B*). This characteristic suggests mitochondrial shortening, swelling, and structural changes triggered by decreased ΔΨm and increased permeability of the inner mitochondrial membrane. 3D‐rendered images of 1,25(OH)_2_D‐treated cells revealed rough, fragmented surfaces (i.e., structural changes) of individual mitochondria treated for 24 hours (Fig. 7 *B*, lower panel). The reversible changes of mitochondria from the tubular to condensed or fragmented conformations is the classic response of loss of ATP synthesis by OXPHOS.^(^ ^53^ ^)^

In EM studies, vehicle‐treated cells exhibited tubular mitochondria; however, individual cristae were hardly discernible, suggestive of deranged mitochondrial respiration^(^ ^54^ ^)^ (Fig. 7*C*, red arrows). Furthermore, some mitochondria were in various stages of membrane fusion/fission as marked by “tethered” structures indicative of dynamic remodeling (Fig. 7*C*, blue arrow). 1,25(OH)_2_D treatment increased the size of mitochondria and generated mitochondria with discernible cristae (Fig. 7*D*, *E*), which may reflect partial prevention of cristolysis. The mitochondria also contained electron‐lucent cavities, not vacuoles,^(^ ^55^ ^)^ consistent with the ring‐shaped structures observed in the IF studies (Fig. 7*B*, white arrow) and may represent interorganellar connections commonly observed in normal cells.^(^ ^56^ ^)^

### 3.8 1,25(OH) _2_D regulation of mitochondrial biogenesis mediates DDIT4/REDD1 availability and mTOR function in the cytoplasm

**Fig 8:**
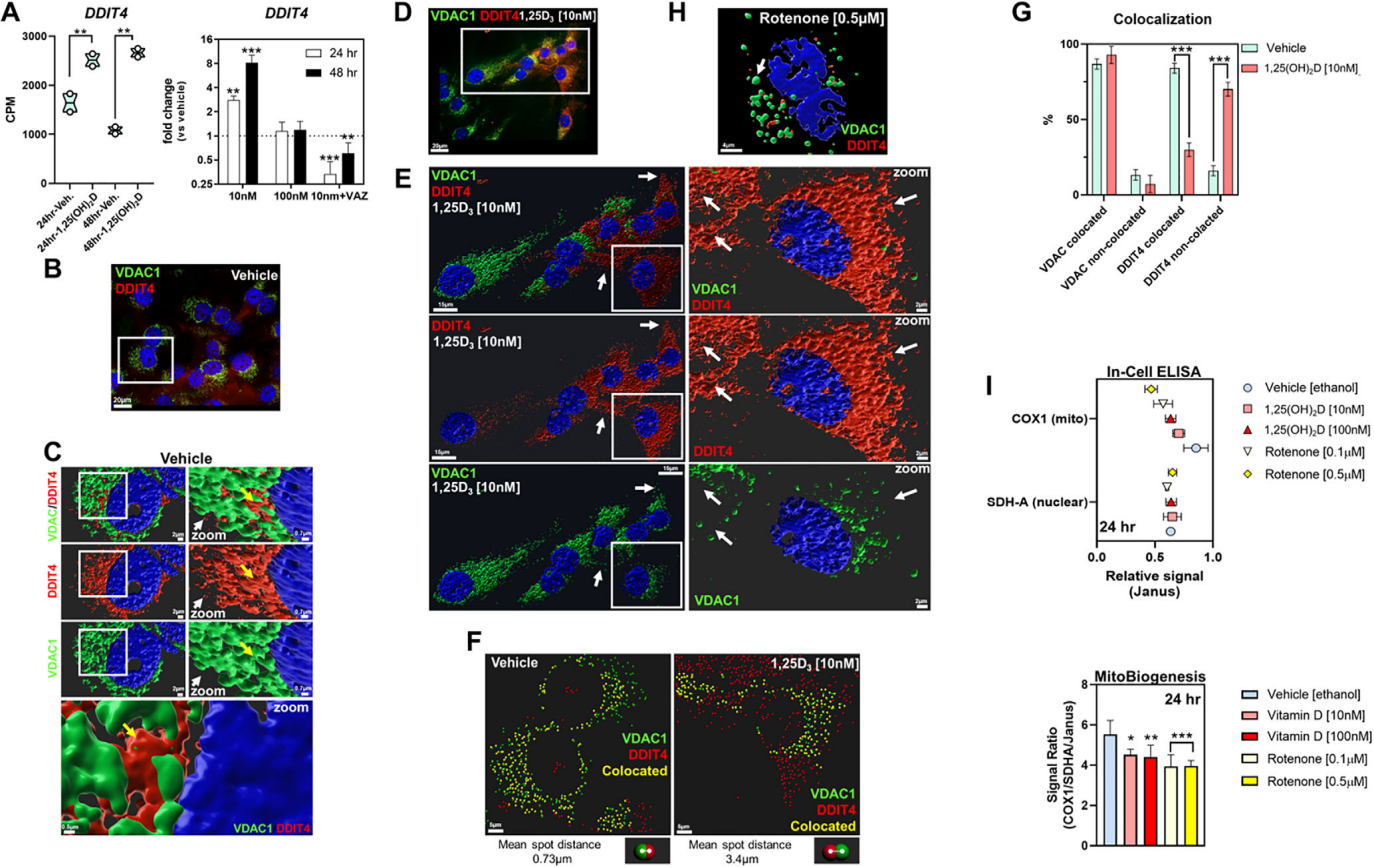
1,25(OH)_2_D regulation of mitochondrial biogenesis and DDIT4/REDD1 cytoplasmic availability. (*A*) DDIT4 transcript levels after vitamin D treatment of MG‐63 cells. The left panel depicts the RNAseq data whereby a two‐way ANOVA was performed with Bonferroni's multiple comparisons test using the counts per million (CPM) values (*n* = 2 samples/condition). The *p* value summaries are depicted as ^**^ *p* ≤ 0.01. The right panel depicts the real‐time PCR validation at two different durations and 1,25(OH)_2_D concentrations. VAZ was used as a VDR‐specific inhibitor. The data are presented as mean ± SEM error bars (*n* = 3 samples/condition); ^***^ *p* ≤ 0.001 and ^**^ *p* ≤ 0.01 (one‐way ANOVA with Tukey's multiple comparisons test compared with respective vehicle). (*B*) Immunofluorescence labeling of vehicle‐treated MG‐63 cells for VDAC1 and DDIT4 after 24 hours. (*C*) 3D Imaris rendering of inset in (*B*). The right panel depicts the magnification of the left panel inset. Yellow arrows depict VDAC1‐DDIT4 colocalized mitochondria, while the white arrows depict sparse cytoplasmic DDIT4. The lower panel depicts high magnification of VDAC1‐DDIT4 colocalization. (*D*) Immunofluorescence labeling of 1,25(OH)_2_D treated MG‐63 cells for VDAC1 and DDIT4 after 24 hours. (*E*) 3D Imaris rendering of inset in (*D*). The right panel depicts the magnification of the left panel inset. White arrows focus on positions of DDIT4 expression relative to VDAC1 placement. (*F*) Representative image of VDAC1‐DDIT4 colocalization and separation after 1,25(OH)_2_D treatment for 24 hours using Imaris. Colocalization and separation analysis was performed using the Imaris “spot” tool to designate the distance threshold and the mean distance between the “VDAC” and “DDIT4” colocalized spots. Yellow spots depict colocated elements. Bottom panel depicts the shortest mean spot distances for each treatment conditions across all colocated spots. (*G*) Quantification of VDAC1 colocalization after 1,25(OH)_2_D treatment for 24 hours using Imaris. A two‐way ANOVA test with Sidak's multiple comparisons test was performed between vehicle and treatment data sets using Prism (GraphPad) where the *p* value summaries were depicted as ^***^ *p* ≤ 0.001. Statistical significance was accepted at ^*^ *p* ≤ 0.05. (*I*) Mitochondrial biogenesis and translation assay after 1,25(OH)_2_D treatment for 24 hours in MG‐63 cells. The upper panel depicts the relative signal of COX1 and SDH‐A normalized to Janus. The bottom panel depicts the level of mitochondrial biogenesis and translation based on the signal ratio of measured factors. Data are presented as mean ± SEM error bars (*n* = 5 replicates/condition); ^***^ *p* ≤ 0.001, ^**^ *p* ≤ 0.01, ^*^ *p* ≤ 0.05 (two‐way ANOVA with Tukey's multiple comparisons test compared with vehicle).

Lastly, given the results of our functional annotation analysis and recent findings that certain cells express DDIT4/REDD1 in the mitochondria,^(^ ^57^ ^)^ we focused the remainder of our attention on the role that 1,25(OH)_2_D and DDIT4 play in cancer prevention. DDIT4 is a known tumor suppressor gene predominantly expressed in the cytoplasm under certain stress conditions to function as a potent mTOR inhibitor.^(^ ^58^ ^)^ However, recent findings show that DDIT4 is highly expressed in malignant cancers, leading to poor cancer‐related prognosis in a paradoxical manner,^(^ ^23^, ^44^ ^)^ suggesting that for certain genes the expression profiles cannot be functionally generalized (Supplemental Fig. S3). To help rationalize this paradoxical observation, we investigated DDIT4 cellular flux in MG‐63 cells before and after 1,25(OH)_2_D treatment. First, 1,25(OH)_2_D at 10 nM increased DDIT4 mRNA levels in a time‐and VDR‐dependent manner (Fig. 8*A*). Next, we performed Apotome (Zeiss) structured‐illumination imaging of DDIT4 and VDAC1 within vehicle‐treated MG‐63 cells and found that DDIT4 was exclusively associated with VDAC1‐positive mitochondria (Fig. 8 *B*, yellow arrow). Imaris 3D‐rendering confirmed the proximity of both VDAC1 and DDIT4 within individual mitochondria (Fig. 8*C*), whereby VDAC1‐free DDIT4 expression in the cytoplasm was uncommon (white arrow) (Fig. 8*C*). A more dynamic colocalization pattern was observed after 1,25(OH)_2_D treatment (Fig. 8*D*, *E*). DDIT4 was predominantly expressed in the cytoplasm after 1,25(OH)_2_D treatment (Fig. 8*E*). Interestingly, among the VDAC1‐DDIT4 colocalized mitochondria in 1,25(OH)_2_D‐treated cells, the average spot distance (i.e., the shortest distance between VDAC1 and DDIT4) was significantly increased compared with controls, suggesting the translocation or leaking of DDIT4 into the cytoplasm (Fig. 8*F*, bottom panel). Furthermore, after rotenone treatment, MG‐63 cells contained globular VDAC1‐positive mitochondria as previously noted, as well as a disassociation of DDIT4 from the mitochondria, suggesting a potential role of mitochondrial depolarization (Fig. 8*H*). Nonetheless, the level of colocalized VDAC1‐DDIT4 protein in 1,25(OH)_2_D‐treated cells was significantly decreased compared with vehicle treatment, reflecting the excess of DDIT4 in the cytoplasm (Fig. 8*G*). In contrast, vehicle‐treated MG‐63 cells contained a statistically significant higher percentage of VDAC1‐DDIT4 colocalized mitochondria with lower levels of cytoplasmic DDIT4 (Fig. 8*G*). Given the apparent decrease in the number of mitochondria after 1,25(OH)_2_D treatment, the increase in cytoplasmic DDIT4 protein may occur, in part, due to reduced mitochondrial biogenesis (i.e., mass/content/self‐replication). To address this possibility, we used a duplexing in‐cell ELISA assay that quantifies both mitochondrial (mt) DNA‐ and nuclear (n) DNA‐encoded proteins, COX‐1 and SDHA, respectively, in MG‐63 cells. We observed 1,25(OH)_2_D‐dependent inhibition of mtDNA‐encoded COX‐1 protein relative to nDNA‐encoded SDHA protein by ~20% after 24 hours (Fig. 8*I*). These data suggest that enhanced mitochondrial localization of DDIT4 may help confer the cancer state and that the enhanced cytoplasmic localization and expression of DDIT4 may be a mechanism by which 1,25(OH)_2_D suppresses osteosarcomas.

## 4 Discussion

### 4.1 Relationship between 1,25(OH) _2_D and the metabolic oxidation/reduction reactions of cancerous and non‐cancerous cells

Findings so far in non‐cancerous cells suggest that proper 25(OH)D levels maintain and minimize systemic cellular oxidative stress after the day‐to‐day exposure to damaging agents such as UV sunlight.^(^ ^59^ ^)^ Furthermore, loss of VDR functional studies in human skin keratinocytes show increased mitochondrial membrane potential due to increased transcription of the respiratory chain subunits II and IV of cytochrome c oxidase.^(^ ^60^ ^)^ In addition, the potential for vitamin D_3_ to reduce oxidative damage to DNA has been linked to a clinical trial where vitamin D_3_ supplementation reduced 8‐hydroxy‐2′‐deoxyguanosine, a marker of oxidative damage, in colorectal epithelial crypt cells.^(^ ^61^ ^)^ In other studies, 1,25(OH)_2_D was shown to modulate the expression of select antioxidative genes via nuclear factor erythroid 2‐related factor 2 (NRF2), which is a key transcription factor that can bind to AREs to protect cells against oxidative stress associated with diabetic neuropathy.^(^ ^62^ ^)^ These findings suggest that vitamin D metabolites can regulate the respiratory chain and to modulate ancillary metabolic pathways depending on the cellular context and requirements within stressed non‐cancerous cells.

**Fig 9:**
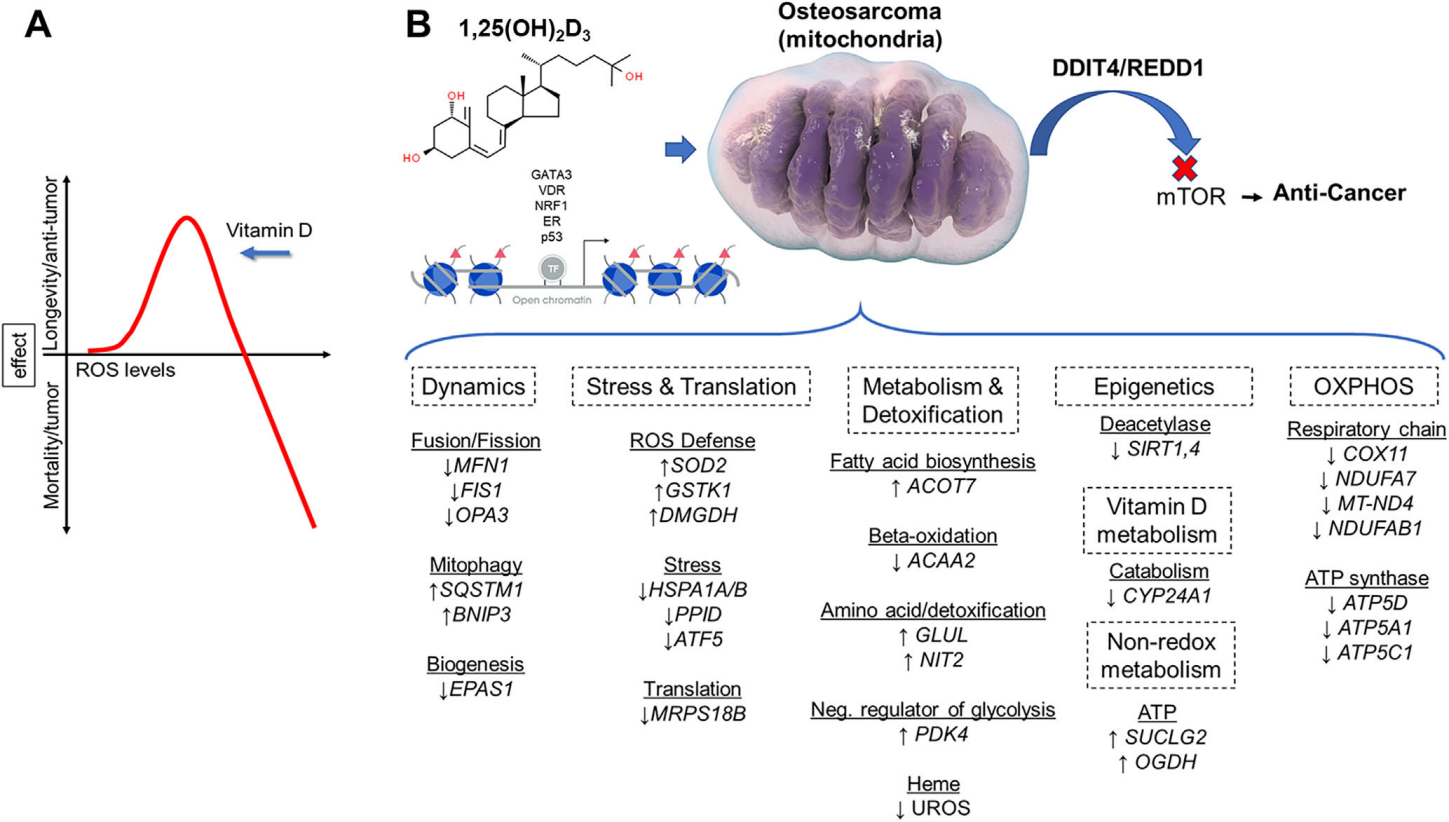
1,25(OH)_2_D regulation of mitochondrial hormesis and functions within MG‐63 osteosarcoma cells. (*A*) 1,25(OH)_2_D suppresses cancer progression by modulating the ROS threshold. 1,25(OH)_2_D‐mediated mitohormesis and ROS production reprograms MG‐63 osteosarcoma cells to enforce stress tolerance and growth inhibition to improve health outcomes. (*B*) 1,25(OH)_2_D modulates chromatin assembly and mitochondrial metabolism, oxidative stress, and mTOR inhibition via DDIT4/REDD1 localization to dictate its overall anticancer response. Red triangles represent acetylation.

Our findings in cancer cells show that 1,25(OH)_2_D can influence mitochondrial metabolism, structure, and function to dictate its anticancer effects, which may also intimately involve extra‐mitochondrial organelles such as the ER (Figs. 3 and 9). Membrane potential is directly related to the activity of mitochondria, with more activity correlated with higher stress levels. Our findings show that there is lower mitochondria activity through the depolarization of the mitochondrial membrane after 1,25(OH)_2_D treatment, hence less stress and ROS production. 1,25(OH)_2_D decreased the mitochondrial membrane potential to a level sufficient for cells to survive but insufficient to generate mt ROS, unlike other mitochondrial depolarizers such as hydrogen peroxide.^(^ ^63^, ^64^ ^)^ Mitochondrial depolarization aims to reverse the inner membrane potential to generate minimal ATP with lower mt ROS, which is associated with increased longevity as observed in long‐lived naked mole rats and bats.^(^ ^65^ ^)^ By contrast, in the absence of 1,25(OH)_2_D, MG‐63 osteosarcomas are accompanied by mechanisms that prevent mitochondrial depolarization, resulting in chronic intracellular protein and DNA damage by mt ROS.^(^ ^66^ ^)^ In addition, 1,25(OH)_2_D treatment resulted in mitochondria with “ring‐like” structures, which may represent intracellular organellar connections such as mitochondria‐ER‐associated membranes where the ER has been pulled into the lumen of the mitochondria to facilitate the direct transfer of phospholipids between the ER and mitochondria to help organize and generate cristae.^(^ ^56^ ^)^ It is also possible that 1,25(OH)_2_D may promote the sharing of other interorganellar resources, such as mitochondria‐derived antioxidants or SOD2, to support the stressed ER, that has the potential to produce additional ROS itself. In contrast, untreated MG‐63 cells exhibit a lack of distinct mitochondrial cristae, suggesting defects in the conductivity of structural proteins and stages of replication with constant flux between fusion and fission where the double‐membrane structures are not as well developed. The potential reduction in fusion and fission after 1,25(OH)_2_D treatment may also result in reduced complementation of mitochondrial gene products that occurs to repair damaged mitochondria in untreated cancer cells. That is to say, the reduced fission/fusion may reflect the limited need for organellar quality control, supported by the downregulation of ATF5. Overall, in MG‐63 cancer cells, 1,25(OH)_2_D functions to decrease mt ROS levels, which may also prevent ROS leakage to other cellular compartments to maintain molecular and organellar integrity.

### 4.2 1,25(OH) _2_D and ROS regulation

How 1,25(OH)_2_D reduces mitochondrial ROS levels is unclear and likely involves multiple factors, including cellular detoxification as well as reduced synthesis in the mitochondria via downregulation of the respiratory chain complex subunits. Although we showed that 1,25(OH)_2_D can enhance SOD2 levels, there are other ROS scavenging and degradation systems such as glutathione peroxidase, glutathione reductase, thioredoxin, and catalase, some of which were also regulated by 1,25(OH)_2_D treatment in our studies. Our results also show that 1,25(OH)_2_D can decrease the beta oxidation of fatty acids as an additional means to reduce ROS levels. 1,25(OH)_2_D may also regulate ROS production and turnover in other organelles besides mitochondria. ROS are also produced in the ER through the oxidation of proteins. Although our ER stress results show the activation of the early stage of the UPR after 1,25(OH)_2_D treatment, the more severe consequences of unfolded proteins (e.g., ER‐mediated apoptosis) were not evident, suggesting that 1,25(OH)_2_D may also prevent this from occurring. In addition, 1,25(OH)_2_D induced heme oxygenase‐1 (HMOX1) expression, which is an essential enzyme localized to the plasma membrane and Golgi apparatus for heme catabolism to form biliverdin, carbon monoxide, and ferrous iron.^(^ ^67^ ^)^ Free heme promotes ROS production by damaging DNA, lipids, and proteins. In conjunction, the generation of carbon monoxide may also regulate key anti‐inflammatory cytokines (IL‐10, IL‐1RA, ↑NFKBIA) that may play an anticancer role in MG‐63 cells after 1,25(OH)_2_D treatment.^(^ ^2^ ^)^

### 4.3 1,25(OH) _2_D and ROS consequences

The direct consequences of lowered ROS levels after 1,25(OH)_2_D treatment on intracellular targets and processes in cancer cells are unknown. It is known that at low levels, ROS can act as signaling molecules in various intracellular processes such as migration. At low levels, ROS differentially alters target protein conformation to modulate stability, folding, and activities of enzymes and ensuing phosphorylation cascades. The decrease in ROS production mediated by 1,25(OH)_2_D is likely to also affect downstream cystine oxidation of proteins to regulate, for example, DNA repair and damage, to facilitate the anticancer response. Overall, our results suggest that after 1,25(OH)_2_D treatment, the mitochondria produce low levels of ROS as a new set point that are effectively scavenged by the cancer cells' antioxidant defense system. It is at this new set point that makes mitochondrial ROS an intracellular signaling molecule that does not induce oxidative stress but instead provides a safe “basal” window for redox signaling to alter the cancer cell fate.

Cell studies have shown that ROS can have both inductive and suppressive effects on global transcription as well as epigenetic responses in a highly cell type–specific manner.^(^ ^68^ ^)^ This may be related to the variable epigenetic and ROS‐sensitive and/or insensitive transcription factors or factors that control the availability of antioxidant thiols within a cell. The effects of ROS on epigenetic and genetic mechanisms can involve direct effects that entail modifications of DNA bases and histones or indirect effects on DNA and histone‐modifying enzymes to control cancer development.^(^ ^69^ ^)^ ROS can also affect class III histone deacetylases (HDACs) called sirtuins (SIRTs). Unlike class I, II, and IV HDACs that use metals as cofactors, SIRTs require NAD^+^ as a functional cofactor, thus making this enzyme sensitive to metabolic and redox changes to relay cellular stress in the form of histone modifications and changes in gene expression.^(^ ^70^ ^)^ In our study, 1,25(OH)_2_D treatment resulted in the downregulation of the mitochondrial SIRT1/4 deacetylases in MG‐63 cells, which may signify a decoupling of lysine deacetylation with NAD+ hydrolysis and PDK4‐acetly‐CoA (histone acetylation) to promote gene expression. Tumor studies have shown that SIRT4 has both oncogenic and tumor‐suppressive activities in cancer depending on the experimental conditions.^(^ ^71^ ^)^ In the context of 1,25(OH)_2_D signaling and concomitant ROS reduction, SIRT1/4 downregulation may help create an epigenomic landscape and balance to facilitate 1,25(OH)_2_D‐specific anticancer transcriptional responses and genomic stability.

### 4.4 1,25(OH) _2_D and stress tolerance and metabolic responses

Unchallenged protein misfolding can elicit cell death, while low levels of stress may be beneficial to cells by eliciting an adaptive UPR.^(^ ^30^ ^)^ Furthermore, the beneficial effects of mild stress on aging and longevity have been studied in experimental animals, whereby mild dietary stress by way of dietary restriction without malnutrition delays age‐related physiological changes and extends the life span. Importantly, animal studies have also demonstrated that mild dietary stress can prevent or lessen the severity of cancer.^(^ ^72^ ^)^ Recent findings using the model organism, *Caenorhabditis elegans*, showed that 1,25(OH)_2_D can promote longevity by enhancing proteostasis,^(^ ^73^ ^)^ which may be akin to our findings of mitochondrial proteostasis and reduced biogenesis in MG‐63 cells. These findings suggest that 1,25(OH)_2_D may mimic a metabolic state induced by dietary restriction and/or mild UPR to improve the life span and anticancer effects. Indeed, our previous studies showed that 1,25(OH)_2_D treatment was comparable to serum starvation of cultured osteoblasts, where suppression of the mTOR pathway was identified as a common feature and known also to be involved in life span expansion in mice when inhibited with rapamycin.^(^ ^74^ ^)^ Furthermore, our RNAseq and ATACseq motif analysis revealed associations with hypoxia, suggesting that 1,25(OH)_2_D may promote tumor starvation by inhibiting vascular perfusion less the negative effects of elevated ROS. Also, 1,25(OH)_2_D can promote mitochondrial depolarization, which is coupled to the availability of glucose or creatine, akin to dietary restriction to support sufficient mitochondrial ATP. These observations can also be metabolically linked to the increase in PDK4 we observed after vitamin D treatment. PDK4 is increased during hibernation/starvation and helps to decrease metabolism and conserve glucose by reducing its conversion to acetyl‐CoA for ATP production.^(^ ^75^ ^)^

Our model suggests that 1,25(OH)_2_D changes the metabolism of cancer cells from being responsive to stress to that of tolerant of stress that involves ER/mitohormetic processes with overall ROS reduction (Figs. 3 and 9). There is recent precedence for this model in the natural immunometabolism setting involving microbial‐macrophage interactions.^(^ ^76^ ^)^ Timblin and colleagues showed that modulation of initial elevated antimicrobial ROS levels within macrophages involves ROS defense strategies as well as metabolic shifts toward non‐oxidative energy metabolism, resulting in a reduction of ROS levels for macrophages to survive and function. Our model similarly shows a parallel paradigm enforced by 1,25(OH)_2_D on the dysregulated metabolism of MG‐63 cancer cells. Co‐opting this stress tolerance response identified in this study by 1,25(OH)_2_D may be a future strategy to consider toward cancer therapy. Importantly, we identified key 1,25(OH)_2_D‐mediated metabolic enzymes that regulate fluxes of small compounds to provide the appropriate basal substrates for cell structure and energy production within dysfunctional osteosarcoma cells. For example, 1,25(OH)_2_D upregulated DMGDH, whereby it acts as an antioxidant when its enzymatic byproduct, dimethylglycine, is used to support the one‐carbon (1‐C) metabolism toward cytosolic NADPH production.^(^ ^35^ ^)^ Importantly, increased DMGDH levels are linked to hepatocellular carcinoma suppression.^(^ ^36^ ^)^ Furthermore, 1,25(OH)_2_D also positively regulates succinyl‐CoA synthase, which facilitates the coupling of succinyl‐CoA synthesis and hydrolysis to substrate level phosphorylation of ADP to ATP.^(^ ^43^ ^)^ The significance of this finding is that despite mitochondrial depolarization and OXPHOS inhibition after 1,25(OH)_2_D treatment, the cell can generate sufficient ATP via non‐redox metabolism independent of mitochondrial electron acceptors to support anticancer biological activities, including survival.

### 4.5 Linking 1,25(OH) _2_D regulation of DDIT4/REDD1 to mitochondria and cancer biology

In the physiological setting, DDIT4 is highly expressed in the cell cytoplasm under stress conditions such as hypoxia, cigarette smoke,^(^ ^77^ ^)^ and UV‐induced DNA damage to function as a potent mTOR inhibitor to suppress cell proliferation and growth, while promoting autophagic processes instead. DDIT4 is also highly expressed in malignant cancers,^(^ ^23^, ^44^ ^)^ despite its known mTOR‐inhibiting properties, suggesting that some cancers have evolved mechanisms to resist DDIT4, which may also antagonize antitumor therapies. For example, a meta‐analysis of individual cancer data sets using gene expression profiling interactive analysis (GEPIA) shows that DDIT4 mRNA expression is significantly increased in numerous tumor tissues such as cervical squamous cell carcinoma (CESC)^(^ ^23^ ^)^ (Supplemental Fig. S3); however, no data on osteosarcoma are currently available. We use GEPIA to further determine the overall cancer survival for CESC based on *DDIT4* gene expression levels. DDIT4 levels were normalized for relative comparison between a housekeeping gene, *ACTB*, and the *VDR* gene. Using the log‐rank test (Mantel‐Cox test) for hypothesis evaluation, the hazard ratio (HR) and the 95% confidence interval (CI) information associated with both gene normalization comparisons suggest a significant association with decreased survival of patients with elevated DDIT4 levels (*p* = 0.0019 and 0.039 and HR = 2.1 and 1.6). The *VDR* relative comparison resulted in a higher *p* value and lower HR, suggesting direct regulation of DDIT4 levels by vitamin D across individuals. This association of decreased survival for high DDIT4 cohorts was observed for many other cancer types besides CESC presented in GEPIA, suggesting elevated DDIT4 is associated with poor prognosis and a vitamin D component.

In line with the findings from GEPIA, our findings in MG‐63 cancer cells show that the mitochondria and their biogenic state can dictate DDIT4 cellular localization pattern and function. In contrast to MG‐63 cancer cells, our previous findings using normal primary osteoblasts showed a robust cytoplasmic expression pattern of DDIT4 under basal settings,^(^ ^22^ ^)^ which suggests a DDIT4 dichotomy between normal and cancer states. Currently, it is unknown if DDIT4 mitochondrial sequestration and biogenesis are a generalized feature of most cancer cell types, and it is likewise unknown how 1,25(OH)_2_D can regulate DDIT4 organellar sequestration and functional outcomes in those cancer cell types. Interestingly, we used an in silico mitochondria targeting sequence (MTS) predictor and identified a putative MTS only in the n‐terminus of DDIT4 that contains a cysteine residue in the cleavage domain (Supplemental Fig. S3). This suggests that 1,25(OH)_2_D, through its effects on ROS production, may regulate DDIT4 interactions via reactive cysteines with the mitochondria. Given the unknown function of DDIT4 in the mitochondria of MG‐63 cells, future studies will focus on better understanding its role in the regulation of cell metabolism and mitochondrial biogenesis in the context of 1,25(OH)_2_D treatment and oxidative signaling.

## Disclosures

All authors state that they have no conflicts of interest.

### Peer Review

The peer review history for this article is available at https://publons.com/publon/10.1002/jbm4.10572.

## Supporting information


**Supplemental Table S1.** Human Real‐Time PCR and Endpoint Primer Sets

**Supplemental Fig. S1.** Correlation matrix of top 75% of RNAseq transcripts. (*A*) Hierarchical clustering tree. The tree generated using genes with maximum expression level at the top 75%. (*B*) Pearson's correlation coefficients across all data sets. (*C*) Principal component analysis using first and second principal components indicates substantial differences in genes induced by 1,25(OH)_2_D treatment. For example, the first principal component explains 38% of the variance. There is little variation among replicates across all data sets. Plots using multidimensional scaling (MDS) and t‐SNE show a similar distribution of our replicate samples.

**Supplemental Fig. S2.** Hierarchical and K‐means clustering of RNAseq data sets. (*A*) Visualization of the relationships/correlations among enriched GO terms using hierarchical clustering tree using iDEP. For the tree construction, we first measured the distance among the GO terms by the percentage of overlapped genes. (*B*) For K‐means clustering, we used the dimension reduction algorithm t‐SNE to map the top 2000 most variable genes, and then examined the distribution to help choose the number of clusters in K‐means. For heatmap generation, we normalized by gene mean center and then mapped back to the GO terms to further generate tables and cluster tree.

**Supplemental Fig. S3.** DDIT4 in cancer and mitochondria. (*A*) Gene expression profiling interactive analysis (GEPIA), a meta‐analysis of individual cancer data sets, shows that DDIT4 mRNA expression is significantly increased in numerous tumor tissues such as adrenocortical carcinoma, cervical squamous cell carcinoma (CESC), head and neck squamous cell carcinoma, kidney renal papillary cell carcinoma, acute myeloid leukemia, lung adenocarcinoma, mesothelioma, and pancreatic adenocarcinoma. No data on osteosarcoma are available through GEPIA. (*B*) GEPIA was used to determine the overall cancer survival for CESC based on *DDIT4* gene expression levels. DDIT4 levels were normalized for relative comparison between a housekeeping gene (*ACTB*) and the *VDR* gene. Using the log‐rank test (Mantel‐Cox test) for hypothesis evaluation, the hazard ratio (HR) and the 95% confidence interval information were included in the survival plots for the high‐ and low‐expressing cohorts. (*C*) In silico approach to identify putative mitochondria targeting sequences in the proximal region of the human DDIT4 protein (UniProt: Q9NX09) using MitoMiner (https://mitominer.mrc-mbu.cam.ac.uk/release-4.0). Based on the amino acid sequence of DDIT4, MitoMiner predicted a mitochondrial targeting sequence with an overall probability score of 0.716 along with the prediction of a cleavage sequence in the N‐terminal region.

## Data Availability

All data generated during and/or analyzed during the current study are available from the corresponding author on reasonable request.

## References

[1] Frezza C . Metabolism and cancer: the future is now. Br J Cancer. 2020;122(2):133‐135. 31819199 10.1038/s41416-019-0667-3 PMC6978144

[2] Bouillon R , Carmeliet G , Verlinden L , et al. Vitamin D and human health: lessons from vitamin D receptor null mice. Endocr Rev. 2008;29(6):726‐776. 18694980 10.1210/er.2008-0004 PMC2583388

[3] Takeyama K , Kitanaka S , Sato T , Kobori M , Yanagisawa J , Kato S . 25‐Hydroxyvitamin D3 1alpha‐hydroxylase and vitamin D synthesis. Science. 1997;277(5333):1827‐1830. 9295274 10.1126/science.277.5333.1827

[4] Bacchetta J , Sea JL , Chun RF , et al. Fibroblast growth factor 23 inhibits extrarenal synthesis of 1,25‐dihydroxyvitamin D in human monocytes. J Bone Miner Res. 2013;28(1):46‐55. 22886720 10.1002/jbmr.1740 PMC3511915

[5] Bacchetta J , Zaritsky JJ , Lisse TS , et al. Vitamin D as a new regulator of iron metabolism: vitamin D suppresses hepcidin in vitro and in vivo. Nephrol Dial Transpl. 2012;27:29‐30.

[6] Bacchetta J , Zaritsky JJ , Sea JL , et al. Suppression of iron‐regulatory hepcidin by vitamin D. J Am Soc Nephrol. 2014;25(3):564‐572. 24204002 10.1681/ASN.2013040355 PMC3935584

[7] Zhou R , Chun RF , Lisse TS , et al. Vitamin D and alternative splicing of RNA. J Steroid Biochem. 2015;148:310‐317. 10.1016/j.jsbmb.2014.09.025 PMC4361308 25447737

[8] Zhou R , Park JW , Chun RF , et al. Concerted effects of heterogeneous nuclear ribonucleoprotein C1/C2 to control vitamin D‐directed gene transcription and RNA splicing in human bone cells. Nucleic Acids Res. 2017;45(2):606‐618. 27672039 10.1093/nar/gkw851 PMC5314791

[9] Lisse TS , Adams JS , Hewison M . Vitamin D and microRNAs in bone. Crit Rev Eukaryot Gene Expr. 2013;23(3):195‐214. 23879537 10.1615/critreveukaryotgeneexpr.2013007147 PMC3857713

[10] Lisse TS , Saini V , Zhao H , Luderer HF , Gori F , Demay MB . The vitamin D receptor is required for activation of cWnt and hedgehog signaling in keratinocytes. Mol Endocrinol. 2014;28(10):1698‐1706. 25180455 10.1210/me.2014-1043 PMC4179637

[11] Zhao H , Rieger S , Abe K , Hewison M , Lisse TS . DNA damage‐inducible transcript 4 is an innate surveillant of hair follicular stress in vitamin D receptor knockout mice and a regulator of wound re‐epithelialization. Int J Mol Sci. 2016;17(12):1984. 10.3390/ijms17121984 PMC5187784 27898044

[12] Uchitomi R , Oyabu M , Kamei Y . Vitamin D and sarcopenia: potential of vitamin D supplementation in sarcopenia prevention and treatment. Nutrients. 2020;12(10):3189. 10.3390/nu12103189 PMC7603112 33086536

[13] Wei Z , Yoshihara E , He N , et al. Vitamin D switches BAF complexes to protect beta cells. Cell. 2018;173(5):1135‐49 e15. 29754817 10.1016/j.cell.2018.04.013 PMC5987229

[14] Lisse TS . Vitamin D regulation of a SOD1‐to‐SOD2 antioxidative switch to prevent bone cancer. Appl Sci‐Basel. 2020;10(7):2554‐2567.

[15] Manson JE , Cook NR , Lee IM , et al. Vitamin D supplements and prevention of cancer and cardiovascular disease. N Engl J Med. 2019;380(1):33‐44. 30415629 10.1056/NEJMoa1809944 PMC6425757

[16] Zhang Y , Fang F , Tang J , et al. Association between vitamin D supplementation and mortality: systematic review and meta‐analysis. BMJ. 2019;366:4673. 10.1136/bmj.l4673 PMC6689821 31405892

[17] Feldman D , Krishnan AV , Swami S , Giovannucci E , Feldman BJ . The role of vitamin D in reducing cancer risk and progression. Nat Rev Cancer. 2014;14(5):342‐357. 24705652 10.1038/nrc3691

[18] Keum N , Giovannucci E . Vitamin D supplements and cancer incidence and mortality: a meta‐analysis. Br J Cancer. 2014;111(5):976‐980. 24918818 10.1038/bjc.2014.294 PMC4150260

[19] Chandler PD , Chen WY , Ajala ON , et al. Effect of vitamin D3 supplements on development of advanced cancer: a secondary analysis of the VITAL randomized clinical trial. JAMA Netw Open. 2020;3(11):e2025850. 33206192 10.1001/jamanetworkopen.2020.25850 PMC7675103

[20] Du J , Cieslak JA 3rd , Welsh JL , Sibenaller ZA , et al. pharmacological ascorbate radiosensitizes pancreatic cancer. Cancer Res. 2015;75(16):3314‐3326. 26081808 10.1158/0008-5472.CAN-14-1707 PMC4537815

[21] Marampon F , Gravina GL , Festuccia C , et al. Vitamin D protects endothelial cells from irradiation‐induced senescence and apoptosis by modulating MAPK/SirT1 axis. J Endocrinol Invest. 2016;39(4):411‐422. 26335302 10.1007/s40618-015-0381-9

[22] Lisse TS , Liu T , Irmler M , et al. Gene targeting by the vitamin D response element binding protein reveals a role for vitamin D in osteoblast mTOR signaling. FASEB J. 2011;25(3):937‐947. 21123297 10.1096/fj.10-172577 PMC3042839

[23] Tang Z , Li C , Kang B , Gao G , Li C , Zhang Z . GEPIA: a web server for cancer and normal gene expression profiling and interactive analyses. Nucl Acids Res. 2017;45(W1):W98‐W102. 28407145 10.1093/nar/gkx247 PMC5570223

[24] Afgan E , Baker D , Batut B , et al. The galaxy platform for accessible, reproducible and collaborative biomedical analyses: 2018 update. Nucl Acids Res. 2018;46(W1):W537‐W544. 29790989 10.1093/nar/gky379 PMC6030816

[25] Yoon, S‐B , Park, Y‐H , Choi, S‐A & et al (2019) Real‐time PCR quantification of spliced X‐box binding protein 1 (XBP1) using a universal primer method. PLoS ONE, 14(7), e0219978. 10.1371/journal.pone.0219978 31329612 PMC6645673

[26] Tokuumi Y . Correlation between the concentration of 1,25 alpha dihydroxyvitamin D3 receptors and growth inhibition, and differentiation of human osteosarcoma cells induced by vitamin D3. Nihon Seikeigeka Gakkai Zasshi. 1995;69(4):181‐190. 7782656

[27] Borowicz S , Van Scoyk M , Avasarala S , et al. The soft agar colony formation assay. J Visual Exp. 2014;(92):e51998. 25408172 10.3791/51998 PMC4353381

[28] Dauletbaev N , Herscovitch K , Das M , et al. Down‐regulation of IL‐8 by high‐dose vitamin D is specific to hyperinflammatory macrophages and involves mechanisms beyond up‐regulation of DUSP1. Br J Pharmacol. 2015;172(19):4757‐4771. 26178144 10.1111/bph.13249 PMC4594277

[29] Roche J . The epithelial‐to‐mesenchymal transition in cancer. Cancers (Basel). 2018;10(2):361‐373. 10.3390/cancers10020052 PMC5836084 29462906

[30] Mollereau B , Manie S , Napoletano F . Getting the better of ER stress. J Cell Commun Sig. 2014;8(4):311‐321. 10.1007/s12079-014-0251-9 PMC4390799 25354560

[31] Mark KA , Dumas KJ , Bhaumik D , et al. Vitamin D promotes protein homeostasis and longevity via the stress response pathway genes skn‐1, ire‐1, and xbp‐1. Cell Rep. 2016;17(5):1227‐1237. 27783938 10.1016/j.celrep.2016.09.086 PMC5689451

[32] Sasagawa S , Nishimura Y , Okabe S , et al. Downregulation of GSTK1 is a common mechanism underlying hypertrophic cardiomyopathy. Front Pharmacol. 2016;7:162. 27378925 10.3389/fphar.2016.00162 PMC4905960

[33] Fiorese CJ , Schulz AM , Lin YF , Rosin N , Pellegrino MW , Haynes CM . The transcription factor ATF5 mediates a mammalian mitochondrial UPR. Curr Biol. 2016;26(15):2037‐2043. 27426517 10.1016/j.cub.2016.06.002 PMC4980197

[34] Rath S , Sharma R , Gupta R , et al. MitoCarta3.0: an updated mitochondrial proteome now with sub‐organelle localization and pathway annotations. Nucl Acids Res. 2021;49(D1):D1541‐D1547. 33174596 10.1093/nar/gkaa1011 PMC7778944

[35] Ducker GS , Rabinowitz JD . One‐carbon metabolism in health and disease. Cell Metab. 2017;25(1):27‐42. 27641100 10.1016/j.cmet.2016.08.009 PMC5353360

[36] Liu G , Hou G , Li L , Li Y , Zhou W , Liu L . Potential diagnostic and prognostic marker dimethylglycine dehydrogenase (DMGDH) suppresses hepatocellular carcinoma metastasis in vitro and in vivo. Oncotarget. 2016;7(22):32607‐32616. 27119355 10.18632/oncotarget.8927 PMC5078037

[37] Rosca MG , Vazquez EJ , Chen Q , Kerner J , Kern TS , Hoppel CL . Oxidation of fatty acids is the source of increased mitochondrial reactive oxygen species production in kidney cortical tubules in early diabetes. Diabetes. 2012;61(8):2074‐2083. 22586586 10.2337/db11-1437 PMC3402323

[38] Lin CH , Chung MY , Chen WB , Chien CH . Growth inhibitory effect of the human NIT2 gene and its allelic imbalance in cancers. FEBS J. 2007;274(11):2946‐2956. 17488281 10.1111/j.1742-4658.2007.05828.x

[39] Park CB , Asin‐Cayuela J , Camara Y , et al. MTERF3 is a negative regulator of mammalian mtDNA transcription. Cell. 2007;130(2):273‐285. 17662942 10.1016/j.cell.2007.05.046

[40] Krick S , Shi S , Ju W , et al. Mpv17l protects against mitochondrial oxidative stress and apoptosis by activation of Omi/HtrA2 protease. Proc Natl Acad Sci USA. 2008;105:14106‐14111. 18772386 10.1073/pnas.0801146105 PMC2529330

[41] Min Z , Gao J , Yu Y . The roles of mitochondrial SIRT4 in cellular metabolism. Front Endocrinol (Lausanne). 2018;9:783. 30666234 10.3389/fendo.2018.00783 PMC6330279

[42] Piantadosi CA , Withers CM , Bartz RR , et al. Heme oxygenase‐1 couples activation of mitochondrial biogenesis to anti‐inflammatory cytokine expression. J Biol Chem. 2011;286(18):16374‐16385. 21454555 10.1074/jbc.M110.207738 PMC3091243

[43] Chinopoulos C , Seyfried TN . Mitochondrial substrate‐level phosphorylation as energy source for glioblastoma: review and hypothesis. ASN Neuro. 2018;10:1759091418818261. 30909720 10.1177/1759091418818261 PMC6311572

[44] Ding F , Gao F , Zhang S , Lv X , Chen Y , Liu Q . A review of the mechanism of DDIT4 serve as a mitochondrial related protein in tumor regulation. Sci Prog. 2021;104(1):36850421997273. 33729069 10.1177/0036850421997273 PMC10455034

[45] Lisse TS , Hewison M , Adams JS . Hormone response element binding proteins: novel regulators of vitamin D and estrogen signaling. Steroids. 2011;76(4):331‐339. 21236284 10.1016/j.steroids.2011.01.002 PMC3042887

[46] Pike JW , Meyer MB . The vitamin D receptor: new paradigms for the regulation of gene expression by 1,25‐dihydroxyvitamin D(3). Endocrinol Metab Clin N Am. 2010;39(2):255‐269. 10.1016/j.ecl.2010.02.007 PMC2879406 20511050

[47] Heinz S , Benner C , Spann N , et al. Simple combinations of lineage‐determining transcription factors prime cis‐regulatory elements required for macrophage and B cell identities. Mol Cell. 2010;38(4):576‐589. 20513432 10.1016/j.molcel.2010.05.004 PMC2898526

[48] Ho IC , Pai SY . GATA‐3—not just for Th2 cells anymore. Cell Mol Immunol. 2007;4(1):15‐29. 17349208

[49] Cancer Genome Atlas Network. Comprehensive molecular portraits of human breast tumours. Nature. 2012;490(7418):61‐70. 23000897 10.1038/nature11412 PMC3465532

[50] Biswas M , Chan JY . Role of Nrf1 in antioxidant response element‐mediated gene expression and beyond. Toxicol Appl Pharmacol. 2010;244(1):16‐20. 19665035 10.1016/j.taap.2009.07.034 PMC2837788

[51] Cao L , Bu R , Oakley JI , Kalla SE , Blair HC . Estrogen receptor‐beta modulates synthesis of bone matrix proteins in human osteoblast‐like MG63 cells. J Cell Biochem. 2003;89(1):152‐164. 12682916 10.1002/jcb.10486

[52] Li JM , Zhou H , Cai Q , Xiao GX . Role of mitochondrial dysfunction in hydrogen peroxide‐induced apoptosis of intestinal epithelial cells. World J Gastroenterol. 2003;9(3):562‐567. 12632519 10.3748/wjg.v9.i3.562 PMC4621583

[53] Rossignol R , Gilkerson R , Aggeler R , Yamagata K , Remington SJ , Capaldi RA . Energy substrate modulates mitochondrial structure and oxidative capacity in cancer cells. Cancer Res. 2004;64(3):985‐993. 14871829 10.1158/0008-5472.can-03-1101

[54] Moscheni C , Malucelli E , Castiglioni S , et al. 3D quantitative and ultrastructural analysis of mitochondria in a model of doxorubicin sensitive and resistant human colon carcinoma cells. Cancers (Basel). 2019;11(9):1254. 10.3390/cancers11091254 PMC6769783 31461915

[55] Lauber JK . Retinal pigment epithelium: ring mitochondria and lesions induced by continuous light. Curr Eye Res. 1982;2(12):855‐862. 7187642 10.3109/02713688209020022

[56] Moltedo O , Remondelli P , Amodio G . The mitochondria‐endoplasmic reticulum contacts and their critical role in aging and age‐associated diseases. Front Cell Dev Biol. 2019;7:172. 31497601 10.3389/fcell.2019.00172 PMC6712070

[57] Sofer A , Lei K , Johannessen CM , Ellisen LW . Regulation of mTOR and cell growth in response to energy stress by REDD1. Mol Cell Biol. 2005;25(14):5834‐5845. 15988001 10.1128/MCB.25.14.5834-5845.2005 PMC1168803

[58] Lisse TS , Hewison M . Vitamin D: a new player in the world of mTOR signaling. Cell Cycle. 2011;10(12):1888‐1889. 21558808 10.4161/cc.10.12.15620 PMC3154412

[59] Vishlaghi N , Lisse TS . Exploring vitamin D signalling within skin cancer. Clin Endocrinol (Oxf). 2020;92(4):273‐281. 31889334 10.1111/cen.14150

[60] Consiglio M , Destefanis M , Morena D , et al. The vitamin D receptor inhibits the respiratory chain, contributing to the metabolic switch that is essential for cancer cell proliferation. PLoS One. 2014;9(12):e115816. 25546457 10.1371/journal.pone.0115816 PMC4278832

[61] Fedirko V , Bostick RM , Long Q , et al. Effects of supplemental vitamin D and calcium on oxidative DNA damage marker in normal colorectal mucosa: a randomized clinical trial. Cancer Epidemiol Biomark Prevent. 2010;19(1):280‐291. 10.1158/1055-9965.EPI-09-0448 PMC2805163 20056649

[62] Forster RE , Jurutka PW , Hsieh JC , et al. Vitamin D receptor controls expression of the anti‐aging klotho gene in mouse and human renal cells. Biochem Biophys Res Commun. 2011;414(3):557‐562. 21982773 10.1016/j.bbrc.2011.09.117 PMC3209523

[63] Lisse TS , King BL , Rieger S . Comparative transcriptomic profiling of hydrogen peroxide signaling networks in zebrafish and human keratinocytes: implications toward conservation, migration and wound healing. Sci Rep. 2016;6:20328. 26846883 10.1038/srep20328 PMC4742856

[64] Lisse TS , Rieger S . IKKalpha regulates human keratinocyte migration through surveillance of the redox environment. J Cell Sci. 2017;130(5):975‐988. 28122935 10.1242/jcs.197343 PMC5358334

[65] Grasso D , Zampieri LX , Capeloa T , Van de Velde JA , Sonveaux P . Mitochondria in cancer. Cell Stress. 2020;4(6):114‐146. 32548570 10.15698/cst2020.06.221 PMC7278520

[66] Xu L , Sun Z , Wei X , et al. The inhibition of MARK2 suppresses cisplatin resistance of osteosarcoma stem cells by regulating DNA damage and repair. J Bone Oncol. 2020;23:100290. 32368441 10.1016/j.jbo.2020.100290 PMC7184251

[67] Dunn LL , Kong SMY , Tumanov S , et al. Hmox1 (heme oxygenase‐1) protects against ischemia‐mediated injury via stabilization of HIF‐1alpha (hypoxia‐inducible factor‐1alpha). Arterioscler Thromb Vasc Biol. 2021;41(1):317‐330. 33207934 10.1161/ATVBAHA.120.315393

[68] Niu Y , DesMarais TL , Tong Z , Yao Y , Costa M . Oxidative stress alters global histone modification and DNA methylation. Free Radic Biol Med. 2015;82:22‐28. 25656994 10.1016/j.freeradbiomed.2015.01.028 PMC4464695

[69] Whetstine JR , Nottke A , Lan F , et al. Reversal of histone lysine trimethylation by the JMJD2 family of histone demethylases. Cell. 2006;125(3):467‐481. 16603238 10.1016/j.cell.2006.03.028

[70] Hwang JW , Yao H , Caito S , Sundar IK , Rahman I . Redox regulation of SIRT1 in inflammation and cellular senescence. Free Radic Biol Med. 2013;61:95‐110. 23542362 10.1016/j.freeradbiomed.2013.03.015 PMC3762912

[71] Huang G , Zhu G . Sirtuin‐4 (SIRT4), a therapeutic target with oncogenic and tumor‐suppressive activity in cancer. Onco Targets Ther. 2018;11:3395‐3400. 29928130 10.2147/OTT.S157724 PMC6001835

[72] Brandhorst S , Longo VD . Fasting and caloric restriction in cancer prevention and treatment. Recent Results Cancer Res. 2016;207:241‐266. 27557543 10.1007/978-3-319-42118-6_12 PMC7476366

[73] Salminen A , Kaarniranta K . ER stress and hormetic regulation of the aging process. Ageing Res Rev. 2010;9(3):211‐217. 20416402 10.1016/j.arr.2010.04.003

[74] Harrison DE , Strong R , Sharp ZD , et al. Rapamycin fed late in life extends lifespan in genetically heterogeneous mice. Nature. 2009;460(7253):392‐395. 19587680 10.1038/nature08221 PMC2786175

[75] Buck MJ , Squire TL , Andrews MT . Coordinate expression of the PDK4 gene: a means of regulating fuel selection in a hibernating mammal. Physiol Genom. 2002;8(1):5‐13. 10.1152/physiolgenomics.00076.2001 11842126

[76] Timblin GA , Tharp KM , Ford B , et al. Mitohormesis reprogrammes macrophage metabolism to enforce tolerance. Nat Metab. 2021;3(5):618‐635. 34031590 10.1038/s42255-021-00392-w PMC8162914

[77] Yoshida T , Mett I , Bhunia AK , et al. Rtp801, a suppressor of mTOR signaling, is an essential mediator of cigarette smoke‐induced pulmonary injury and emphysema. Nat Med. 2010;16(7):767‐773. 20473305 10.1038/nm.2157 PMC3956129

