# Supplementary material for: Vitamin D Modulation of Mitochondrial Oxidative Metabolism and mTOR Enforces Stress Adaptations and Anticancer Responses
Source: JBMR Plus. 2021 Dec 1;6(1):e10572. doi: 10.1002/jbm4.10572 (PMC8771003; doi:10.1002/jbm4.10572)
Supplement: Supplementary file 1 — Supplemental Table S1. Human Real‐Time PCR and Endpoint Primer Sets [file JBM4-6-e10572-s009.docx]

Title: Vitamin D modulation of mitochondrial oxidative metabolism and mTOR enforces stress adaptations and anti-cancer responses

Authors: Mikayla Quigley^1,6^, Sandra Rieger^1,7^, Enrico Capobianco^2^, Zheng Wang^3^, Hengguang Zhao^4^, Martin Hewison^5^, Thomas S. Lisse^1,7*^

Affiliations: ^1^University of Miami, Biology Department

1301 Memorial Drive, Cox Science Center, Coral Gables, Florida 33146 USA.

^2^Institute for Data Science and Computing, Coral Gables, Florida 33146 USA.

^3^Department of Computer Science, Coral Gables, Florida 33146 USA.

^4^Department of Dermatology, The First Affiliated Hospital of Chongqing Medical University, Chongqing 400016, China.

^5^Institute of Metabolism and Systems Research, University of Birmingham, Birmingham, B15 2TT, United Kingdom.

^6^Dana Farber Cancer Institute, Boston, MA 02215 USA.

^7^Sylvester Comprehensive Cancer Center, Miller School of Medicine, University of Miami, Miami, Florida 33136 USA.

^*^Correspondence:

Thomas Lisse Ph.D.

Department of Biology, The University of Miami

tom.lisse@miami.edu

305-284-3957

Keywords: Osteosarcoma, cancer, tumor, vitamin D, vitamin D deficiency, vitamin D receptor, metabolism, VDR, ROS, mitochondria, MG-63, SOD, SOD1, SOD2, stress, bone, osteoblast, CYP24A1, DDIT4, REDD1

Competing interests: Authors have nothing to declare.

Funding: Supported by Grant # IRG-17-183-16 from the American Cancer Society, and from the Sylvester Comprehensive Cancer Center at the Miller School of Medicine, University of Miami

**Supplemental Table**

Supplemental Table S1. Human real-time PCR and end-point primer sets

| **Target Gene** | **Forward (5’-3’)** | **Reverse (5’-3’)** |
| --- | --- | --- |
| ACTB | GCAAAGACCTGTACGCCAAC | ACATCTGCTGGAAGGTGGAC |
| CYP24A1 | TGGCTTCAGGAGAAGGAAAA | ACCAGGGTGCCTGAGTGTAG |
| ATF5 | GGCTCCCTATGAGGTCCTTG | CCATAGCTTCCAGGTCAGGT |
| DNM1L | TGCAAAGGATCATTCAGCAC | TCATTAGCCCACAAGCATCA |
| FIS1 | GGAGGACCTGCTGAAGTTTG | ACGGCCAGGTAGAAGACGTA |
| MFN1 | ACGCCAGATAATGCATCACA | TGGTCCAGCTCAGTCTTTCA |
| MFN2 | CATGGGCATTCTTGTTGTTG | TGGAGCCAGTGTAGCTGATG |
| DDIT4 | CCTGGACAGCAGCAACAGT | TACCAACTGGCTAGGCATCA |
| TIMM22 | GTCCAGCCAAGAGTGAGGAG | TCTTTTGCAGTCGGTGTACG |
| P62 (SQSTM1) | CCAGCACAGAGGAGAAGAGC | GACGGGTCCACTTCTTTTGA |
| PPID/CYPD | CGACTTCACCAACCACAATG | CTCTTTGACGTGACCGAACA |
| GAPDH (ER QPCR) | Accatcttccaggagcgaga | Accatcttccaggagcgaga |
| 18srRNA (ER QPCR) | Aaacggctaccacatccaag | Gctggaattaccgcggct |
| uXBP1 (ER qPCR) | cagactacgtgcacctctgc | ctgggtccaagttgtccagaat |
| sXBP1 (ER qPCR) | gctgagtccgcagcaggt | ctgtccaagttgtccagaat |
| tXBP1 (ER qPCR) | tgaaaaacagagtagcagctcaga | cccaagcgctgtcttaactc |
| u/sXBP1 (eP) | ggtctgctgagtccgcagca | aagggaggctggtaaggaac |
| BIP/HSPA5A | Cgaggaggaggacaagaagg | caccttgaacggcaagaact |

EP (endpoint)
