# Supplementary figures and images for: Vitamin D Modulation of Mitochondrial Oxidative Metabolism and mTOR Enforces Stress Adaptations and Anticancer Responses
Source: JBMR Plus. 2021 Dec 1;6(1):e10572. doi: 10.1002/jbm4.10572 (PMC8771003; doi:10.1002/jbm4.10572)

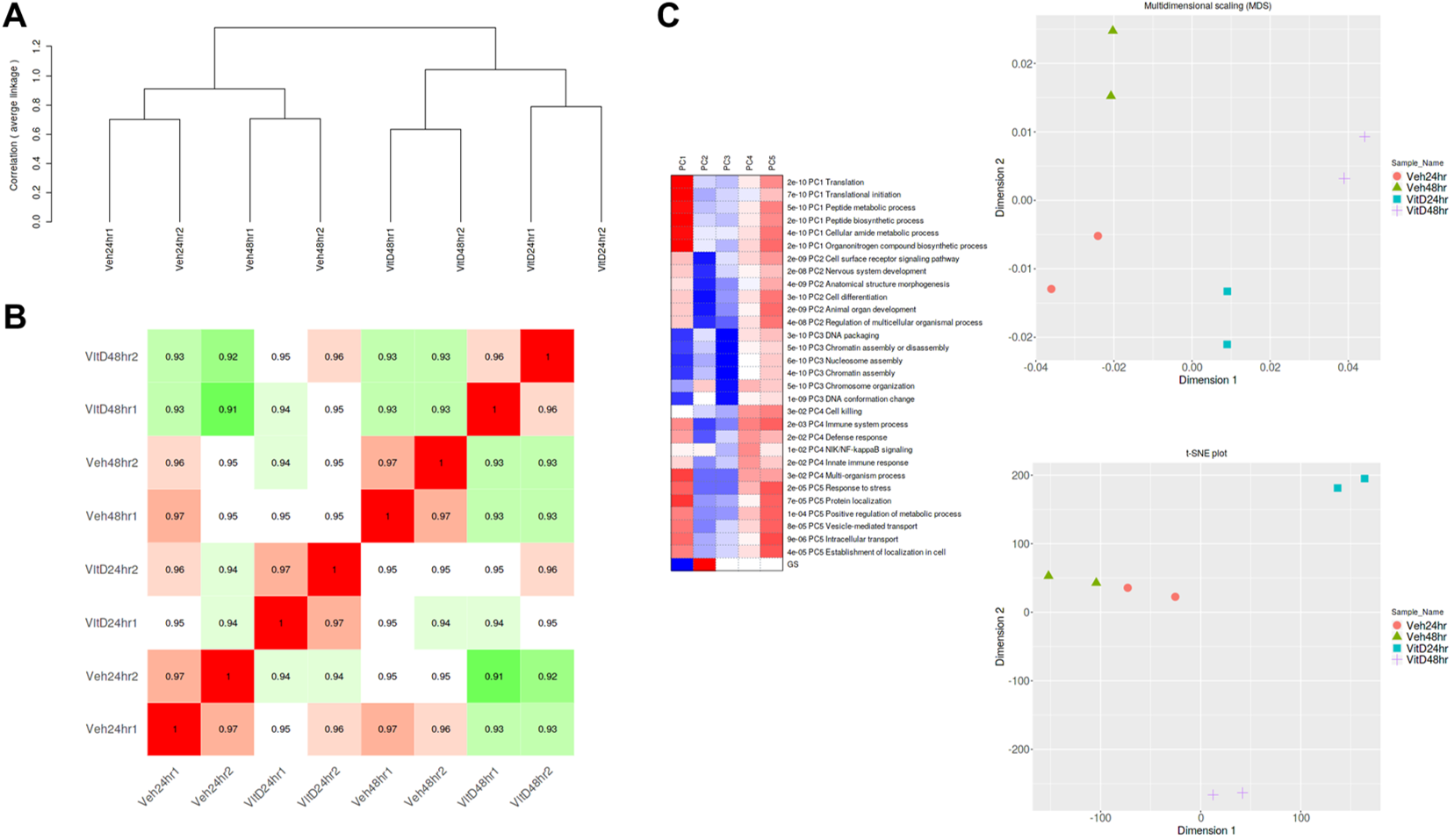

Supplement: Supplementary file 2 — Supplemental Fig. S1. Correlation matrix of top 75% of RNAseq transcripts. (A) Hierarchical clustering tree. The tree generated using genes with maximum expression level at the top 75%. (B) Pearson's correlation coefficients across all data sets. (C) Principal component analysis using first and second principal components indicates substantial differences in genes induced by 1,25(OH)2D treatment. For example, the first principal component explains 38% of the variance. There is little variation among replicates across all data sets. Plots using multidimensional scaling (MDS) and t‐SNE show a similar distribution of our replicate samples. [file JBM4-6-e10572-s008.tif]

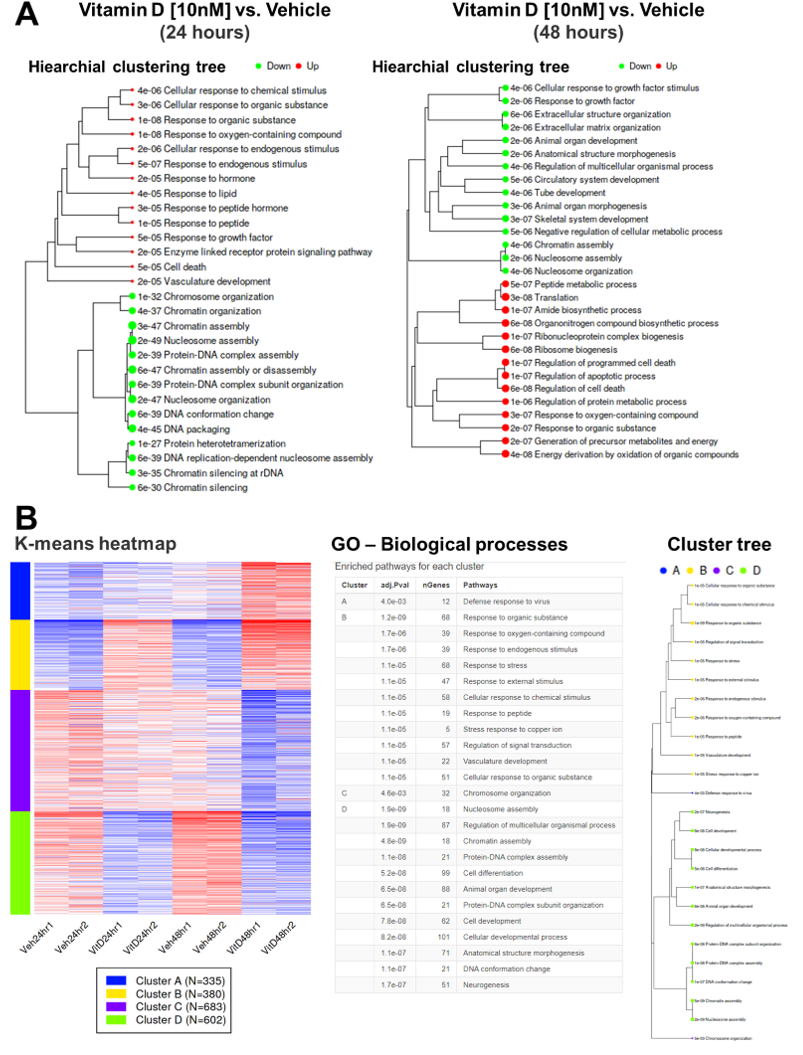

Supplement: Supplementary file 3 — Supplemental Fig. S2. Hierarchical and K‐means clustering of RNAseq data sets. (A) Visualization of the relationships/correlations among enriched GO terms using hierarchical clustering tree using iDEP. For the tree construction, we first measured the distance among the GO terms by the percentage of overlapped genes. (B) For K‐means clustering, we used the dimension reduction algorithm t‐SNE to map the top 2000 most variable genes, and then examined the distribution to help choose the number of clusters in K‐means. For heatmap generation, we normalized by gene mean center and then mapped back to the GO terms to further generate tables and cluster tree. [file JBM4-6-e10572-s014.tif]

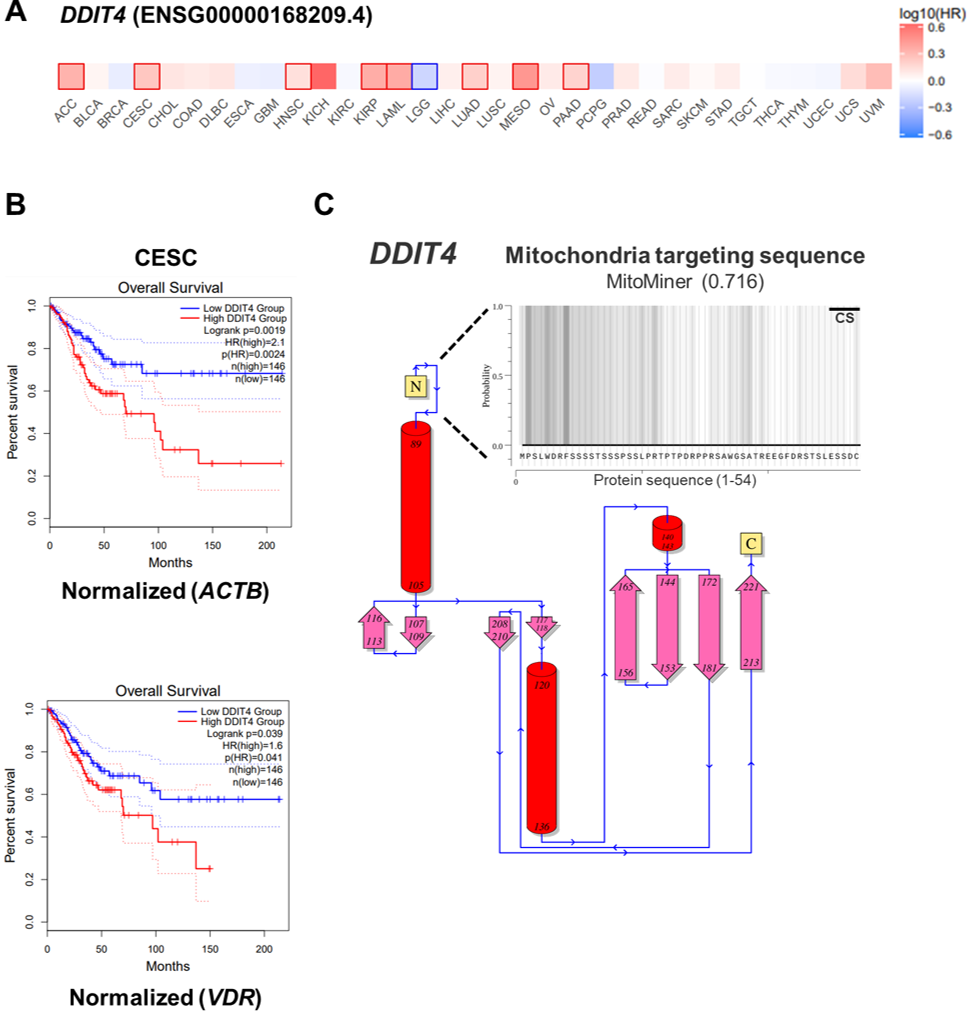

Supplement: Supplementary file 4 — Supplemental Fig. S3. DDIT4 in cancer and mitochondria. (A) Gene expression profiling interactive analysis (GEPIA), a meta‐analysis of individual cancer data sets, shows that DDIT4 mRNA expression is significantly increased in numerous tumor tissues such as adrenocortical carcinoma, cervical squamous cell carcinoma (CESC), head and neck squamous cell carcinoma, kidney renal papillary cell carcinoma, acute myeloid leukemia, lung adenocarcinoma, mesothelioma, and pancreatic adenocarcinoma. No data on osteosarcoma are available through GEPIA. (B) GEPIA was used to determine the overall cancer survival for CESC based on DDIT4 gene expression levels. DDIT4 levels were normalized for relative comparison between a housekeeping gene (ACTB) and the VDR gene. Using the log‐rank test (Mantel‐Cox test) for hypothesis evaluation, the hazard ratio (HR) and the 95% confidence interval information were included in the survival plots for the high‐ and low‐expressing cohorts. (C) In silico approach to identify putative mitochondria targeting sequences in the proximal region of the human DDIT4 protein (UniProt: Q9NX09) using MitoMiner (https://mitominer.mrc-mbu.cam.ac.uk/release-4.0). Based on the amino acid sequence of DDIT4, MitoMiner predicted a mitochondrial targeting sequence with an overall probability score of 0.716 along with the prediction of a cleavage sequence in the N‐terminal region. [file JBM4-6-e10572-s004.tif]
